# Local two-sided bounds for eigenvalues of self-adjoint operators

**DOI:** 10.1007/s00211-016-0822-1

**Published:** 2016-07-09

**Authors:** G. R. Barrenechea, L. Boulton, N. Boussaïd

**Affiliations:** 1 Department of Mathematics and Statistics, University of Strathclyde, 26 Richmond Street, Glasgow, G1 1XH Scotland; 2 Department of Mathematics and Maxwell Institute for Mathematical Sciences, Heriot-Watt University, Edinburgh, EH14 4AS UK; 3 Laboratoirde de Mathématiques, UFR Sciences et Techniques, Université de Franche-Comté, 16, route de Gray, Besançon, 25030 France

**Keywords:** 65M60, 65L60, 65L15, 65N12

## Abstract

We examine the equivalence between an extension of the Lehmann–Maehly–Goerisch method developed a few years ago by Zimmermann and Mertins, and a geometrically motivated method developed more recently by Davies and Plum. We establish a general framework which allows sharpening various previously known results in these two settings and determine explicit convergence estimates for both methods. We demonstrate the applicability of the method of Zimmermann and Mertins by means of numerical tests on the resonant cavity problem.

## Introduction

In this work we study in close detail the equivalence between two pollution-free techniques for numerical computation of eigenvalue bounds for general self-adjoint operators: a method considered a few years ago by Zimmermann and Mertins [35], and a method developed more recently by Davies and Plum [23]. These two methods are pollution-free by construction and have been proven to provide reliable numerical approximations.

The approach of Zimmermann and Mertins is built on an extension of the Lehmann–Maehly–Goerisch method [4, 26, 33] and it has proven to be highly successful in various concrete applications. These include the computation of bounds for eigenvalues of the radially reduced magnetohydrodynamics operator [15, 35], the study of complementary eigenvalue bounds for the Helmholtz equation [6] and the calculation of sloshing frequencies [4, 5].

The method of Davies and Plum on the other hand, is based on a notion of approximated spectral distance and is highly geometrical in character. Its original formulation dates back to [21–23] but it is yet to be tested properly on models of dimension larger than one.

In this work we follow the analysis conducted in [23, Section 6] where the equivalence of both these techniques was formulated in a precise manner. Our main goal is two-fold. On the one hand we examine more closely the nature of this equivalence by considering multiple eigenvalues. On the other hand we determine sharp estimates for both methods. These results include convergence and error estimates for both the eigenvalues and associated eigenfunctions. We finally illustrate the applicability of the method of Zimmermann and Mertins using the Maxwell eigenvalue problem as benchmark.

### Context, scope and contribution of the present work

The computational approach considered in this work has a “local” character, in the sense that a shift parameter should be set before hand. The methods derived from this approach only provide information about the spectrum in a vicinity of this parameter, in similar fashion as the Galerkin method gives information only about the eigenvalues below the bottom of the essential spectrum. They give upper bounds for the eigenvalues to the right of the parameter and lower bounds for the eigenvalues to the left of it.

The method of Davies and Plum primarily relies on the geometrical properties of a notion of approximated spectral distance. We introduce this notion in Sect. 3. Our Proposition 2 was first formulated in [21, theorems 3 and 4]. These statements played a fundamental role in the proof of [23, Theorem 11] which provided crucial connections with the method of Zimmermann and Mertins. In Proposition 5 and Corollary 6 we establish an extension of [21, theorems 3 and 4] allowing multiple eigenvalues. These rely on convexity results due to Danskin (see Lemma 4 and [8, Theorem D1]) and they are of fundamental importance in various parts of our analysis.

Our Lemma 9 follows the original [23, Theorem 11] and its proof involves very similar arguments. In conjunction with Corollary 6, it leads to an alternative proof of [35, Theorem 1.1] which includes multiplicity counting. The latter is the central statement of what we call the method of Zimmermann and Mertins. This alternative derivation of the method is formulated in our main Theorem 10 and Corollary 11.

Theorems 13 and 14, and Corollary 15, are precise formulations of convergence in the setting of the method of Davies and Plum. The two theorems differ from one another in that a higher order of approximation occurs when the shift parameter is away from the spectrum. In Theorem 16 we show that, remarkably, the method of Zimmermann and Mertins always renders the higher order of approximation as a consequence of Corollary 15. This is, for instance, in great agreement with the results presented in [34], which compare the errors in Lehmann–Goerisch and Rayleigh–Ritz bounds (see also [28], where convergence of iterative solvers is studied).

In Proposition 7 we establish upper bounds for error estimates for eigenfunctions in terms of spectral gaps. This statement is related to similar results of Weinberger [32] and Trefftz [30]. See also [33, Chapter 5]. The precise connection between Proposition 7 and all these results is unclear at present and will be examined elsewhere.

The model of the isotropic resonant cavity that we consider in Sect. 6 has been well-documented to render spectral pollution when the classical Galerkin method and finite elements of nodal type are employed for numerical approximation. We show by means of numerical tests that, remarkably, the method of Zimmermann and Mertins provides robust and accurate approximations of the eigenvalues of the Maxwell operator even when implemented on standard Lagrange elements. By construction, this method is free from spectral pollution. A more systematic investigation in this respect with many more numerical tests (including anisotropic media), a convergent algorithm and a reference to a fully reproducible computer code can be found in [3].

Preliminary information on the number of eigenvalues in a given interval, which might or might not be available in practice, allows the determination of enclosures from the one-sided bounds produced by the approaches discussed in this work. Convergence also yields enclosures in suitable asymptotic regimes. The algorithm described in [3] is an example of a concrete realisation of this assertion.

### Outline of the analysis

Section 2 includes the notational conventions and assumptions which will be used throughout this work. Section 3 sets the general framework of approximated spectral distances and their geometrical properties. There we also discuss approximation of eigenspaces with explicit estimates. The method of Zimmermann and Mertins is derived in Sect. 4 and its convergence is established in Sect. 5. These two sections comprise the main contribution of this work. The final Sect. 6 is devoted to illustrating a concrete computational application of the method of Zimmermann and Mertins to the resonant cavity problem.

## Preliminary notation, conventions, and assumptions

Let $A:{\text {D}}(A)\longrightarrow \mathcal {H}$ be a self-adjoint operator acting on a Hilbert space $\mathcal {H}$. Decompose the spectrum of *A* in the usual fashion, as the disjoint union of discrete and essential spectra, $\sigma (A)=\sigma _\mathrm {disc}(A)\cup \sigma _\mathrm {ess}(A)$. Let *J* be any Borel subset of $\mathbb R$. Below the spectral projector associated to *A* is denoted by ${\mathbbm {1} }_{J}(A)=\int _J \mathrm {d}E_\lambda$, so that ${{\mathrm{Tr}}}{\mathbbm {1} }_{J}(A)=\dim {\mathbbm {1} }_{J}(A) \mathcal {H}.$ We write $\mathcal {E}_J(A)=\oplus _{\lambda \in J} \ker (A-\lambda )$ with the convention $\mathcal {E}_\lambda (A)=\mathcal {E}_{\{\lambda \}}(A)$. Generally $\mathcal {E}_J(A)\subseteq {\mathbbm {1} }_{J}(A)\mathcal {H}$, however there is no reason for these two subspaces to be equal except when the spectrum within *J* is only pure point.

Everywhere below $t\in \mathbb R$ will denote a scalar parameter. This is the shift parameter which is intrinsic to the methods.

Let $l_t:{\text {D}}(A)\times {\text {D}}(A)\longrightarrow \mathbb {C}$ be the (not necessarily closed) bi-linear form associated to $(A-t)$,

$$\begin{aligned} l_t(u,w)=\langle (A-t)u,w\rangle \quad \forall u,w\in {\text {D}}(A). \end{aligned}$$

Let $q_t:{\text {D}}(A)\times {\text {D}}(A)\longrightarrow \mathbb {C}$ be the closed bi-linear form

1 $$\begin{aligned} q_t(u,w)=\langle (A-t)u,(A-t)w\rangle \quad \forall u,w\in {\text {D}}(A). \end{aligned}$$

For any $u\in {\text {D}}(A)$ we will constantly refer to the following *t*-dependant semi-norm, which is a norm if *t* is not an eigenvalue,

2 $$\begin{aligned} |u|_t =q_t(u,u)^{1/2}=\Vert (A-t)u\Vert . \end{aligned}$$

By virtue of the min–max principle, $q_t$ characterises the part of the spectrum of the positive operator $(A-t)^2$ which lies near the origin. As we shall see next, this gives rise to a notion of local counting function at *t* for the spectrum of *A*.

Let

3 $$\begin{aligned} \mathfrak {d}_j(t) = \inf _{ \begin{array}{c} \dim V=j\\ V\subset {\text {D}}(A) \end{array}} \sup _{u\in V} \frac{|u|_t}{\Vert u\Vert } \end{aligned}$$

so that $0\le \mathfrak {d}_j(t)\le \mathfrak {d}_{k}(t)$ for $j<k$. Then $\mathfrak {d}_{1}(t)$ is the Hausdorff distance from *t* to $\sigma (A)$,

4 $$\begin{aligned} \mathfrak {d}_1(t)=\min \{|\lambda -t| : \lambda \in \sigma (A)\} = \inf _{u\in {\text {D}}(A)}\frac{|u|_t}{\Vert u\Vert }. \end{aligned}$$

Similarly $\mathfrak {d}_j(t)$ are the distances from *t* to the *j*th nearest point in $\sigma (A)$ counting multiplicity but in a generalised sense. That is, the sequence $(\mathfrak {d}_j(t))_{j\in \mathbb N}$ becomes stationary when it attains the distance from *t* to the essential spectrum. Moreover

$$\begin{aligned} \mathfrak {d}_{j}(t)=\mathfrak {d}_{j-1}(t) \iff \left\{ \begin{array}{ll} \text {either} &{} \dim \mathcal {E}_{[t-\mathfrak {d}_{j-1}(t),t+\mathfrak {d}_{j-1}(t)]}(A)> j-1 \\ \text {or} &{} t\pm \mathfrak {d}_{j-1}(t)\in \sigma _\mathrm {ess}(A). \end{array} \right. \end{aligned}$$

Set

$$\begin{aligned} \delta _j(t)={\text {dist}}\left[ t,\sigma (A){\setminus }\left\{ t\pm \mathfrak {d}_k(t)\right\} _{k=1} ^j\right] . \end{aligned}$$

Let

$$\begin{aligned} \mathfrak {n}^-_j(t)= & {} \sup \{s<t:{{\mathrm{Tr}}}{\mathbbm {1} }_{(s,t]}(A)\ge j\} \quad \text {and} \\ \mathfrak {n}^+_j(t)= & {} \inf \{s>t:{{\mathrm{Tr}}}{\mathbbm {1} }_{[t,s)}(A)\ge j\}, \end{aligned}$$

conveying that $\mathfrak {n}^-_j(t)=-\infty$ whenever ${{\mathrm{Tr}}}{\mathbbm {1} }_{(-\infty ,t]}(A)< j$ and $\mathfrak {n}^+_j(t)=+\infty$ whenever ${{\mathrm{Tr}}}{\mathbbm {1} }_{[t,+\infty )}(A)< j$. Then $\mathfrak {n}^\mp _j(t)$ is the *j*th point in $\sigma (A)$ to the left$(-)$/right$(+)$ of *t* counting multiplicities. Here $t\in \sigma (A)$ is allowed and neither *t* nor $\mathfrak {n}_j^{\mp }(t)$ have to be isolated from the rest of $\sigma (A)$. Without further mention, all the statements below regarding bounds on $\mathfrak {n}_j^\mp (t)$ will be immediate and useless in either of these two cases and so will not be considered in the proofs.

Set

$$\begin{aligned} \nu _j^-(t)&=\sup \{ s<t : {{\mathrm{Tr}}}{\mathbbm {1} }_{(s,t)}(A)\ge j\} \quad \text {and} \\ \nu _j^+(t)&=\inf \{ s>t : {{\mathrm{Tr}}}{\mathbbm {1} }_{(t,s)}(A)\ge j\}. \end{aligned}$$

These are the spectral points of *A* which are strictly to the left and strictly to the right of *t* respectively. The inequality $\nu _j^\pm (t)\not = \mathfrak {n}^\pm _j(t)$ only occurs when *t* is an eigenvalue.

Everywhere below $\mathcal {L}\subset {\text {D}}(A)$ will be a (trial) subspace of dimension $n=\dim \mathcal {L}$. Unless explicitly stated, we will assume the following.

### Assumption 1

The combination of parameter *t* and subspace $\mathcal {L}$ are such that

5 $$\begin{aligned} \mathcal {L}\cap \mathcal {E}_t(A)= \{0\}. \end{aligned}$$

The integer number $m\le n$ will always be chosen such that the following assumption holds true.

### Assumption 2

6 $$\begin{aligned}{}[t-\mathfrak {d}_m(t),t+\mathfrak {d}_m(t)]\cap \sigma (A)\subseteq \sigma _\mathrm {disc}(A). \end{aligned}$$

By virtue of (6), $\delta _j(t)> \mathfrak {d}_j(t)$ for all $j\le m$.

## Approximated local counting functions

In this section we show how to extract certified information about $\sigma (A)$ in the vicinity of *t* from the action of *A* onto $\mathcal {L}$, see [21, Section 3]. For $j\le n$, let

7 $$\begin{aligned} F_j(t) =\min _{ \begin{array}{c} \dim V=j\\ V\subset \mathcal {L} \end{array}} \max _{u\in V} \frac{|u|_t}{\Vert u\Vert }. \end{aligned}$$

Then $0\le F_1(t)\le \cdots \le F_n(t)$ and $F_j(t)\ge \mathfrak {d}_j(t)$ for all $j=1,\ldots ,n$.

As a consequence of the triangle inequality, $F_j$ is a Lipschitz continuous function such that

8 $$\begin{aligned} |F_j(t)-F_j(s)|\le |t-s| \qquad \forall s,t\in \mathbb R\quad \text {and} \quad j=1,\ldots ,n. \end{aligned}$$

Since $[t-\mathfrak {d}_j(t),t+\mathfrak {d}_j(t)]\subseteq [t-F_j(t),t+F_j(t)]$, there are at least *j* spectral points of *A* in the segment $[t-F_j(t),t+F_j(t)]$. As we shall see next, this possibly includes a part of the essential spectrum.

### Lemma 1

For any $$j=1,\ldots ,n$$,

9 $$\begin{aligned} {{\mathrm{Tr}}}{{\mathbbm {1} }}_{[t-F_j(t),t+F_j(t)]}(A) \ge j. \end{aligned}$$

### Proof

Let *B* be a non-negative self-adjoint operator such that $\mathcal {L}\subset {\text {D}}(B)\subset {\text {D}}(B^{1/2})$. Let $b(u)=\langle B^{1/2}u,B^{1/2}u\rangle$ for all $u\in {\text {D}}(B^{1/2})$ be the closure of the quadratic form associated to *B*. Let

$$\begin{aligned} \tilde{\lambda }_j(\mathcal {L})=\min _{ \begin{array}{c} \dim V=j\\ V\subset \mathcal {L} \end{array}} \max _{u\in V} \frac{b(u)}{\Vert u\Vert ^2} \end{aligned}$$

and

$$\begin{aligned} \lambda _j=\inf _{ \begin{array}{c} \dim V=j\\ V\subset {\text {D}}(B^{1/2}) \end{array}} \sup _{u\in V} \frac{b(u)}{\Vert u\Vert ^2}. \end{aligned}$$

We claim that, if $\tilde{\lambda }_j(\mathcal {L})=\lambda _j$, then $\lambda _j$ must be an eigenvalue of *B*. In other words, $\mathcal {E}_{\lambda _j}(B)\ne \{0\}$. Let us firstly verify the validity of this claim.

Suppose that $j=1$. Then

$$\begin{aligned} \lambda _1= \inf _{u\in {\text {D}}(B^{1/2})} \frac{b(u)}{\Vert u\Vert ^2} \end{aligned}$$

is attained by a non-zero vector $v\in \mathcal {L}$. Using the Rayleigh–Ritz principle (see [20, §4.5]), we deduce that $v\in {\text {D}}(B)$ and in fact *v* is an eigenvector associated with $\lambda _1$. This implies the above claim for $j=1$.

Now suppose that $j\ge 2$. We have two possibilities. Either $\tilde{\lambda }_j(\mathcal {L})=\lambda _j$ is in the discrete spectrum of *B* and the claim follows, or it is in the essential spectrum. In the latter case, without loss of generality we can assume that $\tilde{\lambda }_j(\mathcal {L})\not \in \sigma _\mathrm {disc}(B)$ and $\lambda _{j-1}\in \sigma _\mathrm {disc}(A)$. That is, $\lambda _k\in \sigma _\mathrm {disc}(B)$ for any $k\in \{1,\ldots ,j-1\}$ and $\lambda _k=\lambda _j$ for any $k\in \{j,\ldots ,n\}$.

Let

$$\begin{aligned} \mathcal {L}'=\mathcal {L}+\left[ \bigoplus _{k=1}^{j-1} \mathcal {E}_{\lambda _k}(B)\right] . \end{aligned}$$

Then $\tilde{\lambda }_k(\mathcal {L}')=\lambda _k$ for any $k\in \{1,\ldots ,j-1\}$ and

$$\begin{aligned} \lambda _j\le \tilde{\lambda }_j(\mathcal {L}')\le \tilde{\lambda }_j(\mathcal {L}). \end{aligned}$$

But, since $\tilde{\lambda }_j(\mathcal {L})=\lambda _j$, then also $\lambda _j= \tilde{\lambda }_j(\mathcal {L}')$. Now, in the orthogonal decomposition

$$\begin{aligned} \mathcal {L}'=\hat{\mathcal {L}}\oplus \left[ \bigoplus _{k=1}^{j-1} \mathcal {E}_{\lambda _k}(B)\right] , \end{aligned}$$

$\hat{\mathcal {L}}$ is the subspace of $\mathcal {L}'$ orthogonal to $\bigoplus _{k=1}^{j-1} \mathcal {E}_{\lambda _k}(B)$ and it is different from $\mathcal {L}$ in general. For all $u\in \hat{\mathcal {L}},$

$$\begin{aligned} b(u) \ge \lambda _j\Vert u\Vert ^2 \end{aligned}$$

and $$\tilde{\lambda }_1(\hat{\mathcal {L}})=\lambda _j$$. Hence,

$$\begin{aligned} \min _{u\in \hat{\mathcal {L}}}\frac{b(u)}{\Vert u\Vert ^2}= \lambda _j=\min _{u\in {\text {D}}(B^{1/2}{\mathbbm {1} }_{J}(B))}\frac{b(u)}{\Vert u\Vert ^2}. \end{aligned}$$

Thus, from the case $j=1$ already proven, we deduce that $\lambda _j$ is indeed an eigenvalue of *B*. This is the above claim for $j\ge 2$.

We now complete the proof of the lemma. Recall (3) and (7). We have two possibilities, either $F_j(t)=\mathfrak {d}_j(t)$ or $F_j(t)>\mathfrak {d}_j(t)$.

Suppose that $F_j(t)=\mathfrak {d}_j(t)$. From the previous claim for $B=(A-t)^2$ we deduce that

$$\begin{aligned} \mathcal {E}_{\mathfrak {d}_j(t)^2}((A-t)^2)\ne \{0\}. \end{aligned}$$

Hence, according to the Spectral Mapping Theorem, the segment $[t-\mathfrak {d}_j(t),t+\mathfrak {d}_j(t)]$ contains *j* eigenvalues and so

$$\begin{aligned} {{\mathrm{Tr}}}{\mathbbm {1} }_{[t-F_j(t),t+F_j(t)]}(A)= {{\mathrm{Tr}}}{\mathbbm {1} }_{[t-\mathfrak {d}_j(t),t+\mathfrak {d}_j(t)]}(A) \ge j \end{aligned}$$

as needed.

Now suppose that $F_j(t)>\mathfrak {d}_j(t)$. Then $t\mp \mathfrak {d}_j(t)\in [t-F_j(t),t+F_j(t)]$. Moreover, either $t-\mathfrak {d}_j(t)$ or $t+\mathfrak {d}_j(t)$ lies in the essential spectrum and is either isolated from $\sigma (A)$ or is an accumulation point of eigenvalues of *A* or is an endpoint of a segment in $\sigma (A)$. Thus,

$$\begin{aligned} {{\mathrm{Tr}}}{\mathbbm {1} }_{[t-F_j(t),t+F_j(t)]}(A)\ge & {} {{\mathrm{Tr}}}{\mathbbm {1} }_{[t-F_j(t),t-\mathfrak {d}_j(t)]}(A)+{{\mathrm{Tr}}}{\mathbbm {1} }_{[t+\mathfrak {d}_j(t),t+F_j(t)]}(A)\\= & {} \infty \ge j, \end{aligned}$$

and hence once again the conclusion of the lemma is guaranteed. $\square$

By virtue of this lemma, $F_j(t)$ can be regarded as an approximated local counting function for $\sigma (A)$. Moreover, $F_j(t)$ is the *j*th smallest eigenvalue $\mu$ of the non-negative weak problem:

10 $$\begin{aligned} \text {find }(\mu , u)\in [0,\infty )\times \mathcal {L}\! {\setminus }\!\{0\} \quad \text {such that}\quad q_t(u,v) = \mu ^2\langle u,v\rangle \qquad \forall v\in \mathcal {L}. \end{aligned}$$

Hence, we also have the following characterisation,

11 $$\begin{aligned} F_j(t) =\max _{ \begin{array}{c} \dim V=j-1\\ V\subset \mathcal {L} \end{array}} \min _{u\in \mathcal {L}\ominus V} \frac{|u|_t}{\Vert u\Vert }=\max _{ \begin{array}{c} \dim V=j-1\\ V\subset \mathcal {H} \end{array}} \min _{u\in \mathcal {L}\ominus V} \frac{|u|_t}{\Vert u\Vert }. \end{aligned}$$

### Optimal setting for local detection of the spectrum

As we show next, it is possible to detect the spectrum of *A* to the left/right of *t* by means of $F_j$ in an optimal setting. This is a crucial ingredient in the formulation of the strategy proposed in [21–23].

The following statement was first formulated in [21, theorems 3 and 4] and will be sharpened in Corollary 6.

#### Proposition 2

Let $$t^-<t<t^+$$. Then

12 $$\begin{aligned} F_j(t^-)\le t-t^-\Rightarrow & {} t^- - F_j(t^-)\le \mathfrak {n}_j^-(t) \nonumber \\ F_j(t^+)\le t^+-t\Rightarrow & {} t^+ + F_j(t^+)\ge \mathfrak {n}_j^+(t). \end{aligned}$$

Moreover, let $t_1^-<t_2^-<t<t_2^+<t_1^+$. Then

13 $$\begin{aligned} F_j(t_{i}^-)\le t-t_i^-\quad \text {for}\; i=1,2\Rightarrow & {} t_1^- - F_j(t_1^-)\le t_2^- - F_j(t_2^-)\le \mathfrak {n}_j^-(t) \nonumber \\ F_j(t_i^+)\le t_i^+-t \quad \text {for}\; i=1,2\Rightarrow & {} t_1^+ + F_j(t_1^+)\ge t_2^+ + F_j(t_2^+)\ge \mathfrak {n}_j^+(t). \end{aligned}$$

#### Proof

We begin by showing (12). Suppose that $t\ge F_j(t^-)+t^-$. Then

$$\begin{aligned} {{\mathrm{Tr}}}{\mathbbm {1} }_{[t^- -F_j (t^-),t]}(A)\ge j. \end{aligned}$$

Since $\mathfrak {n}_j^-(t)\le \cdots \le \mathfrak {n}_1^-(t)$ are the only spectral points in the segment $[\mathfrak {n}_j^-(t),t]$, then necessarily

$$\begin{aligned} \mathfrak {n}^-_j(t)\in [t^- -F_j (t^-),t]. \end{aligned}$$

The second statement in (12) is shown in a similar fashion and the assertion (13) follows by observing that the maps $t\mapsto t\pm F_j(t)$ are monotonically increasing as a consequence of (8). $\square$

The structure of the trial subspace $\mathcal {L}$ determines the existence of $t^\pm$ satisfying the hypothesis in (12). If we expect to detect $\sigma (A)$ at both sides of *t*, from Poincaré’s Eigenvalue Separation Theorem [9, Theorem III.1.1], a necessary requirement on $\mathcal {L}$ should certainly be the condition

14 $$\begin{aligned} \min _{u\in \mathcal {L}} \frac{\langle Au,u\rangle }{\langle u,u\rangle }<t<\max _{u\in \mathcal {L}} \frac{\langle Au,u\rangle }{\langle u,u\rangle }. \end{aligned}$$

By virtue of Lemmas 8 and 9 below, for $j=1$, the left hand side inequality of (14) implies the existence of $t^-$ and the right hand side inequality implies the existence of $t^+$, respectively.

#### Remark 1

From Proposition 2 it follows that optimal lower bounds for $\mathfrak {n}_j^-(t)$ are achieved by finding $\hat{t}^-_j\le t$, the closest point to *t*, such that $F_j(\hat{t}^-_j)=t-\hat{t}^-_j$. Indeed, by virtue of (13), $t^- - F_j(t^-)\le \hat{t}^-_j - F_j(\hat{t}^-_j)\le \mathfrak {n}_j^-(t)$ for any other $t^-$ as in (12). Similarly, optimal upper bounds for $\mathfrak {n}_j^+(t)$ are found by analogous means. This observation will play a crucial role in Sect. 4.

Proposition 2 is central to the hierarchical method for finding eigenvalue inclusions examined a few years ago in [21, 22]. For fixed $\mathcal {L}$ this method leads to bounds for eigenvalues which are far sharper than those obtained from the obvious idea of estimating local minima of $F_1(t)$. From an abstract perspective, Proposition 2 provides an intuitive insight on the mechanism for determining complementary bounds for eigenvalues. The method proposed in [21–23] is yet to be explored more systematically in a practical setting. However in most circumstances, the technique described in [35], considered in detail in Sect. 4, is easier to implement.

### Geometrical properties of the first approximated counting function

We now determine various geometrical properties of $F_1$ and examine its connection to the spectral distance.

Let $\lambda \in \sigma (A)$ be isolated from the rest of the spectrum. If there exists a non-vanishing $u\in \mathcal {L}\cap \mathcal {E}_\lambda (A)$ (recall Assumption 1), then

$$\begin{aligned} \frac{|u|_s}{\Vert u\Vert }=|\lambda -s|=\mathfrak {d}_1(s) \quad \forall s\in \left[ \lambda -\frac{|\lambda -\nu _1^-(\lambda )|}{2},\lambda +\frac{|\lambda -\nu _1^+(t)|}{2} \right] . \end{aligned}$$

According to the convergence analysis carried out in Sect. 5, the closer $\mathcal {L}$ is to the spectral subspace $\mathcal {E}_\lambda (A)$, the closer $F_1(t)$ is to $\mathfrak {d}_1(t)$ for $t\in (\lambda -\frac{|\lambda -\nu _1^-(\lambda )|}{2},\lambda +\frac{|\lambda -\nu _1^+(\lambda )|}{2 })$. The special case of $\mathcal {L}$ and $\mathcal {E}_\lambda (A)$ having a non-trivial intersection is considered in the following lemma.

#### Lemma 3

For $\lambda \in \sigma (A)$ isolated from the rest of the spectrum, the following statements are equivalent.

- There exists a minimiser $u\in \mathcal {L}$ of the right side of (7) for $j=1$, such that $|u|_t=\mathfrak {d}_1(t)$ for a single $t\in (\lambda -\frac{|\lambda -\nu _1^-(\lambda )|}{2}, \lambda +\frac{|\lambda -\nu _1^+(\lambda )|}{2})$,
- $$F_1(t)=\mathfrak {d}_1(t)$$ for a single $$t\in (\lambda -\frac{|\lambda -\nu _1^-(\lambda )|}{2}, \lambda +\frac{|\lambda -\nu _1^+(\lambda )|}{2})$$,
- $$F_1(s)=\mathfrak {d}_1(s)$$ for all $$s\in [\lambda -\frac{|\lambda -\nu _1^-(\lambda )|}{2},\lambda +\frac{|\lambda -\nu _1^+(\lambda )|}{2}]$$,
- $$\mathcal {L}\cap \mathcal {E}_\lambda (A)\not =\{0\}$$.

#### Proof

Since $\mathcal {L}$ is finite-dimensional, (a) and (b) are equivalent by the definitions of $\mathfrak {d}_1(t)$, $F_1(t)$ and $q_t$. From the paragraph above the statement of the lemma it is clear that (d) $\Rightarrow$ (c) $\Rightarrow$ (b). Since $|u|_t/ \Vert u\Vert$ is the square root of the Rayleigh quotient associated to the operator $(A-t)^2$, the fact that $\lambda$ is isolated combined with the Rayleigh–Ritz principle, gives the implication (a) $\Rightarrow$ (d). $\square$

As there can be a mixing of eigenspaces, it is not possible to replace (b) in this lemma by an analogous statement including $t=\lambda \pm \frac{|\lambda -\nu _1^\pm (\lambda )|}{2}$. If $\lambda '=\lambda +|\lambda -\nu _1^+(\lambda )|$ is an eigenvalue, for example, then $F_1(\frac{\lambda +\lambda '}{2})=\mathfrak {d}_1(\frac{ \lambda +\lambda '}{2})$ ensures that $\mathcal {L}$ contains elements of $\mathcal {E}_\lambda (A) \oplus \mathcal {E}_{\lambda '}(A)$. However it is not guaranteed to contain elements of any of these two subspaces.

### Geometrical properties of the subsequent approximated counting functions

Various extensions of Lemma 3 to the case $j>1$ are possible, however it is difficult to write these results in a neat fashion. Proposition 5 below is one such an extension.

We start presenting a preliminary result needed for its proof. Let $J\subset \mathbb R$ be an open segment. Denote by

$$\begin{aligned} \partial _{t}^{\pm } f(t)= \lim _{\tau \rightarrow 0^+}\pm \frac{f(t\pm \tau )-f(t)}{\tau }, \end{aligned}$$

the one-side derivatives of a function $f:J\longrightarrow \mathbb R$, if they exist. Let $\mathcal {V}$ be a compact topological space. For given $\mathcal {J}:J\times \mathcal {V}\longrightarrow \mathbb R$ we write

$$\begin{aligned} \tilde{\mathcal {J}}(t)=\max _{v\in \mathcal {V}} \mathcal {J}(t,v) \quad \text { and } \quad \tilde{\mathcal {V}}(t)=\left\{ \tilde{v}\in \mathcal {V}: \tilde{\mathcal {J}}(t)=\mathcal {J}(t,\tilde{v})\right\} . \end{aligned}$$

Below we consider an upper semi-continuous function $\mathcal {J}$. Together with the fact that $\mathcal {V}$ is compact, this ensures the existence of $\tilde{\mathcal {J}}(t)$. Using the notation just introduced, we state the following generalization of Danskin’s Theorem, which is a direct consequence of [8, Theorem D1].

#### Lemma 4

If the map $\mathcal {J}$ is upper semi-continuous and $\partial _{t}^{\pm }\mathcal {J}(t,v)$ exist for all $(t,v)\in J\times \mathcal {V}$, then also $\partial _{t}^{\pm }\tilde{\mathcal {J}}(t)$ exist for all $t\in J$ and

15 $$\begin{aligned} \partial _{t}^{\pm }\tilde{\mathcal {J}}(t)=\max _{\tilde{v}\in \tilde{\mathcal {V}}(t)} \partial _{t}^{\pm }\tilde{\mathcal {J}}(t,\tilde{v}). \end{aligned}$$

In the statement of this lemma, note that the left and right derivatives of both $\mathcal {J}$ and $\tilde{\mathcal {J}}$ can be different.

#### Proposition 5

Let $j=1,\ldots ,n$ and $t\in \mathbb R$ be fixed. The next assertions are equivalent.

- $$|F_j(t)-F_j(s)|= |t-s|$$ for some $$s\not =t$$.
- There exists an open segment $J\subset \mathbb R$ containing *t* in its closure, such that
  $$\begin{aligned} |F_j(t)-F_j(s)|= |t-s| \quad \forall s\in \overline{J}. \end{aligned}$$
- There exists an open segment $J\subset \mathbb R$ containing *t* in its closure, such that
  $$\begin{aligned} \forall s\in J,\text { either} \quad \mathcal {L}\cap \mathcal {E}_{s+ F_j(s)}\not =\{0\} \quad \text {or} \quad \mathcal {L}\cap \mathcal {E}_{s- F_j(s)}(A)\not =\{0\}. \end{aligned}$$

#### Proof

$\underline{ \mathrm{(a)} \Rightarrow \mathrm{(b)}}$. Assume (a). Since $r\mapsto r\pm F_j(r)$ are continuous and monotonically increasing, then they have to be constant in the closure of

$$\begin{aligned} J=\{\tau t+(1-\tau )s:0<\tau < 1\}. \end{aligned}$$

This is precisely (b).

$\underline{ \mathrm{(b)} \Rightarrow \mathrm{(c)}}$. Assume (b). Then $s\mapsto F_j(s)$ is differentiable in *J* and its one-sided derivatives are equal to 1 or $-1$ in the whole of this interval. For this part of the proof, we aim at applying (15), in order to get another expression for these derivatives.

Let $\mathcal {F}_j$ be the family of $(j-1)$-dimensional linear subspaces of $\mathcal {L}$. Identify an orthonormal basis of $\mathcal {L}$ with the canonical basis of $\mathbb {C}^n$. Then any other orthonormal basis of $\mathcal {L}$ is represented by a matrix in $\mathrm {O}(n)$, the orthonormal group. By picking the first $(j-1)$ columns of these matrices, we cover all possible subspaces $V\in \mathcal {F}_j$. Indeed we just have to identify $(\underline{v}_1|\cdots |\underline{v}_{j-1})$ for $[\underline{v}_{kl}]_{kl=1}^n \in \mathrm {O}(n)$ with $V=\mathrm {Span}\{\underline{v}_k\}_{k=1}^{j-1}$.

Let

$$\begin{aligned} \mathcal {K}_j=\left\{ (\underline{v}_1,\ldots ,\underline{v}_{j-1}): [\underline{v}_{kl}]_{kl=1}^n \in \mathrm {O}(n) \right\} \subset \underbrace{\mathbb {C}^n\times \cdots \times \mathbb {C}^n}_{j-1}. \end{aligned}$$

Then $\mathcal {K}_j$ is a compact subset in the product topology of the right hand side. According to (11),

$$\begin{aligned} F_j(s)=\max _{(\underline{v}_1,\ldots ,\underline{v}_{j-1})\in \mathcal {K}_j} g(s;\underline{v}_1,\ldots ,\underline{v}_{j-1}) \end{aligned}$$

where

$$\begin{aligned} g(s;\underline{v}_1,\ldots ,\underline{v}_{j-1})=\min _{\begin{array}{c} (a_1,\ldots ,a_{j-1})\in \mathbb {C}^{j-1}\\ \sum |a_k|^2=1 \end{array}} \left| \sum a_k \tilde{v}_k \right| _s. \end{aligned}$$

Here we have used the correspondence between $\underline{v}_k\in \mathbb {C}^{n}$ and $\tilde{v}_k\in \mathcal {L}$ in the orthonormal basis set above. We write

$$\begin{aligned} g(r,V)=g(r;\underline{v}_1,\ldots ,\underline{v}_{j-1}) \quad \text {for} \quad V=\mathrm {Span}\{\tilde{\underline{v}}_k\}_{k=1}^{j-1} \in \mathcal {F}_j. \end{aligned}$$

The map $g:J\times \mathcal {K}_j\longrightarrow \mathbb R^+$ is the minimum of a differentiable function, so the hypotheses of Lemma 4 are satisfied by $\mathcal {J}=-g$. By virtue of (15),

$$\begin{aligned} \partial _{s}^{\pm } g(s,V)=\min _{\begin{array}{c} u\in \mathcal {L}\ominus V, \Vert u\Vert =1\\ |u|_s=g(s,V) \end{array}} \left( \frac{{\text {Re}}l_s(u,u)}{|u|_s}\right) . \end{aligned}$$

As minima of continuous functions, *g*(*s*, *V*) and $\partial _{s}^{\pm }g(s,V)$ are upper semi-continuous. Therefore, a further application of Lemma 4 yields

$$\begin{aligned} \partial _{s}^{\pm } F_j(s)&=\max _{\begin{array}{c} (\underline{v}_1,\ldots ,\underline{v}_{j-1})\in \mathcal {K}_j \\ g(s;\underline{v}_1,\ldots ,\underline{v}_{j-1})=F_j(s) \end{array}} \partial _s^\pm g(s,\underline{v}_1,\ldots ,\underline{v}_{j-1})\\&=\max _{\begin{array}{c} V\in \mathcal {F}_j \\ g(s,V)=F_j(s) \end{array}} \min _{\begin{array}{c} u\in \mathcal {L}\ominus V, \Vert u\Vert =1\\ |u|_s=g(s,V) \end{array}} \left( \frac{{\text {Re}}l_s(u,u)}{|u|_s}\right) . \end{aligned}$$

Now, this shows that

$$\begin{aligned} \left| \max _{\begin{array}{c} V\in \mathcal {F}_j \\ g(s,V)=F_j(s) \end{array}} \min _{\begin{array}{c} u\in \mathcal {L}\ominus V, \Vert u\Vert =1\\ |u|_s=g(s,V) \end{array}} \left( \frac{{\text {Re}}l_s(u,u)}{|u|_s}\right) \right| = 1. \end{aligned}$$

As $\mathcal {L}$ is finite dimensional, there exists a vector $u\in \mathcal {L}$ satisfying $|u|_s=F_j(s)$ such that

$$\begin{aligned} \frac{|{\text {Re}}l_s(u,u)|}{|u|_s}= 1. \end{aligned}$$

Thus $|{\text {Re}}\langle (A-s)u,u\rangle |=\langle (A-s)u,(A-s)u\rangle = F_j(s)$. Hence, according to the “equality” case in the Cauchy–Schwarz inequality, *u* must be an eigenvector of *A* associated with either $s+F_j(s)$ or $s-F_j(s)$. This is precisely (c).

$\underline{\mathrm{(c)} \Rightarrow \mathrm{(a)}}$. Under the condition (c), there exists an open segment $\tilde{J}\subseteq J$, possibly smaller, such that $t\in \overline{\tilde{J}}$ and $F_j(s)=\mathfrak {d}_j(s)$ for all $s\in \tilde{J}$. Since $|\mathfrak {d}_j(s)-\mathfrak {d}_j(r)|=|s-r|$, then either (a) is immediate, or it follows by taking $r\rightarrow t$. $\square$

Proposition 5 leads to the following version of Proposition 2 for *t* an eigenvalue.

#### Corollary 6

Recall Assumption 1. Let $t\in \sigma (A)$ be an eigenvalue of multiplicity *k*. Let $t^-<t<t^+$. If $\mathcal {E}_t(A)\cap \mathcal {L}= \{0\}$, then

16 $$\begin{aligned} F_j(t^-)\le t-t^-\Rightarrow & {} t^- - F_j(t^-)\le \mathfrak {n}_{j+k}^-(t) \nonumber \\ F_j(t^+)\le t^+-t\Rightarrow & {} t^+ + F_j(t^+)\ge \mathfrak {n}_{j+k}^+(t). \end{aligned}$$

#### Proof

According to (9),

$$\begin{aligned} {{\mathrm{Tr}}}{\mathbbm {1} }_{[t^- -F_j (t^-),t^- +F_j (t^-)]}(A)\ge j. \end{aligned}$$

Thus, if $t> F_j(t^-)+t^-$, there is nothing to prove.

Consider now the case $t=F_j(t^-)+t^-$. If there exists $\tau <t^-$ such that $t=F_j(\tau )+\tau$, then (Proposition 5) there exists an open segment $J\subset \mathbb R$ containing $(\tau ,t^-)$ such that

$$\begin{aligned} \forall s\in J,\text { either} \quad \mathcal {L}\cap \mathcal {E}_{s+ F_j(s)}\not =\{0\} \quad \text {or} \quad \mathcal {L}\cap \mathcal {E}_{s- F_j(s)}(A)\not =\{0\}. \end{aligned}$$

From the assumption, it follows that only the second alternative takes place, and necessarily $s - F_j(s)$ is an eigenvalue of *A* for all $s\in (\tau ,t^-)$. Hence, as $s - F_j(s)$ is continuous and $\mathcal {H}$ is separable, this function should be constant in the segment $(\tau ,t^-)$. Moreover, due to monotonicity for any $s\in (\tau ,t^-)$, $s+F_j(s)=t^-$. Hence if $s\in (\tau ,t^-)\mapsto s - F_j(s)$ is constant (equal to some value, say *v*), then *s* is the midpoint between *t* and *v* for any $s\in (\tau ,t^-)$. This contradicts the fact that $\tau \ne t^-$. Hence

$$\begin{aligned} t> F_j(\tau )+\tau , \quad \forall \tau <t^- \end{aligned}$$

and so

$$\begin{aligned} \tau - F_j(\tau )\le \mathfrak {n}_{j+k}^-(t), \end{aligned}$$

for all $\tau <t^-$. By continuity, it then follows that also

$$\begin{aligned} t^- - F_j(t^-)\le \mathfrak {n}_{j+k}^-(t). \end{aligned}$$

The second statement (16) is shown in a similar fashion. $\square$

### Approximated eigenspaces

We conclude this section by showing how to obtain certified information about spectral subspaces.

Our model is the implication (b) $\Rightarrow$ (d) in Lemma 3. In a suitable asymptotic regime for $\mathcal {L}$, the distance between these eigenfunctions and the spectral subspaces of $|A-t|$ in the vicinity of the origin is controlled by a term which is as small as $\mathcal {O}(\sqrt{F_j(t)-\mathfrak {d}_j(t)})$ for $F_j(t)-\mathfrak {d}_j(t)\rightarrow 0$.

The following statement is independent, but it is clearly connected with classical results of Weinberger [32] and Trefftz [30]. Note that a shift parameter can be introduced in Weinberger’s formulation following [4].

#### Proposition 7

Let *m* be as in Assumption 2. Let $t\in \mathbb R$ and $j\in \{1,\ldots ,m\}$ be fixed. Let $\{u_j^t\}_{j=1}^n\subset \mathcal {L}$ be an orthonormal family of eigenfunctions associated to the eigenvalues $\mu =F_j(t)$ of the weak problem (10). Suppose that $F_j(t)-\mathfrak {d}_j(t)$ is small enough so that $0<\varepsilon _j<1$ holds true in the following inductive construction,

$$\begin{aligned} \varepsilon _1= & {} \sqrt{\frac{F_1(t)^2-\mathfrak {d}_1(t)^2}{\delta _1(t)^2-\mathfrak {d}_1(t)^2} } \\ \varepsilon _j= & {} \sqrt{\frac{F_j(t)^2-\mathfrak {d}_j(t)^2}{\delta _j(t)^2-\mathfrak {d}_j(t)^2} +\sum _{k=1}^{j-1} \frac{\varepsilon ^2_k}{1-\varepsilon _k^2} \left( 1+\frac{\mathfrak {d}_j(t)^2-\mathfrak {d}_k(t)^2}{\delta _j(t)^2-\mathfrak {d}_j(t)^2}\right) }. \end{aligned}$$

Then, there exists an orthonormal basis $\{\phi _j^t\}_{j=1}^m$ of $\mathcal {E}_{{[t-\mathfrak {d}_m(t),t+\mathfrak {d}_m(t)]}}(A)$ such that $\phi _j^t\in \mathcal {E}_{\{t- \mathfrak {d}_j(t),t+ \mathfrak {d}_j(t)\}}(A)$,

17 $$\begin{aligned} \Vert u_j^t-\langle u_j^t,\phi _j^t\rangle \phi _j^{t}\Vert\le & {} \varepsilon _j \quad \text {and}\end{aligned}$$

18 $$\begin{aligned} |u_j^t-\langle u_j^t,\phi _j^t\rangle \phi _j^t|_t\le & {} \sqrt{F_j(t)^2-\mathfrak {d}_j(t)^2+ \mathfrak {d}_j(t)^2\varepsilon _j^2}. \end{aligned}$$

#### Proof

As it is clear from the context, in this proof we suppress the index *t* on top of any vector. We write $\Pi _\mathcal {S}$ to denote the orthogonal projection onto the subspace $\mathcal {S}$ with respect to the inner product $\langle \cdot ,\cdot \rangle$.

Let us first consider the case $j=1$. Let $\mathcal {S}_1=\mathcal {E}_{\{t- \mathfrak {d}_1(t),t+ \mathfrak {d}_1(t)\}}\!(A)$ and decompose $u_1=\Pi _{\mathcal {S}_1}u_1+u_1^\perp$ where $u_1^\perp \perp \mathcal {S}_1$. Since *A* is self-adjoint,

19 $$\begin{aligned} F_1(t)^2 =\Vert (A-t)u_1\Vert ^2=\mathfrak {d}_1(t)^2\Vert \Pi _{\mathcal {S}_1} u_1\Vert ^2+\Vert (A-t)u_1^\perp \Vert ^2. \end{aligned}$$

Hence

$$\begin{aligned} F_1(t)^2 \ge \mathfrak {d}_1(t)^2(1-\Vert u_1^\perp \Vert ^2)+\delta _1(t)^{{2}} \Vert u_1^\perp \Vert ^2. \end{aligned}$$

Since $\delta _1(t)> \mathfrak {d}_1(t)$, clearing from this identity $\Vert u_1^\perp \Vert ^2$ yields $\Vert u_1^\perp \Vert \le \varepsilon _1$. Hence $\Vert \Pi _{\mathcal {S}_1}u_1\Vert ^2 \ge 1-\varepsilon _1^2>0$. Let

$$\begin{aligned} \phi _1=\frac{1}{\Vert \Pi _{\mathcal {S}_1}u_1\Vert } {\Pi _{\mathcal {S}_1}u_1} \end{aligned}$$

so that $\Vert \Pi _{\mathcal {S}_1}u_1\Vert =|\langle u_1,\phi _1\rangle |$. Then (17) holds immediately and (18) is achieved by clearing $\Vert (A-t)u_1^\perp \Vert ^2$ from (19). This is the case $j=1$.

Let us now look at the case $j>1$. We define the needed basis, and show (17) and (18), for *j* up to *m* inductively as follows. Set

$$\begin{aligned} \phi _j=\frac{1}{\Vert \Pi _{\mathcal {S}_j} u_j\Vert }\Pi _{\mathcal {S}_j} u_j \end{aligned}$$

where $\mathcal {S}_j=\mathcal {E}_{\{t- \mathfrak {d}_j(t),t+ \mathfrak {d}_j(t)\}}\!(A)\ominus {\text {Span}}\{\phi _l\}_1^{j-1}$ and $\Pi _{\mathcal {S}_j}u_j\not =0$, all this for $1\le j \le k-1$. Assume that (17) and (18) hold true for *j* up to $k-1$. Define $\mathcal {S}_k=\mathcal {E}_{\{t- \mathfrak {d}_k(t),t+ \mathfrak {d}_k(t)\}}\!(A)\ominus {\text {Span}}\{\phi _l\}_1^{k-1}$. We first show that $\Pi _{\mathcal {S}_k}u_k\not =0$, and so we can define

20 $$\begin{aligned} \phi _k=\frac{1}{\Vert \Pi _{\mathcal {S}_k} u_k\Vert }\Pi _{\mathcal {S}_k} u_k \end{aligned}$$

ensuring $\phi _k\perp {\text {Span}}\{\phi _l\}_{l=1}^{k-1}$. After that, we verify the validity of (17) and (18) for $j=k$.

Decompose

$$\begin{aligned} u_k=\Pi _{\mathcal {S}_k}u_k+\sum _{l=k-1}^{1} \langle u_k,\phi _l\rangle \phi _l+u_k^\perp \end{aligned}$$

where $u_k^\perp$ is orthogonal to ${\text {Span}}\{\phi _l\}_{l=1}^{k-1}\oplus \mathcal {S}_k$. Then

$$\begin{aligned} F_k(t)^2&= \mathfrak {d}_k(t)^2\Vert \Pi _{\mathcal {S}_k}u_k \Vert ^2+\sum _{l=k-1}^{1} \mathfrak {d}_l(t)^2|\langle u_k,\phi _l\rangle |^2 + \Vert (A-t)u_k^\perp \Vert ^2 \\&\ge \mathfrak {d}_k(t)^2 \Vert \Pi _{\mathcal {S}_k}u_k \Vert ^2 +\sum _{l=k-1}^{1} \mathfrak {d}_l(t)^2 |\langle u_k,\phi _l\rangle |^2 +\delta _k(t)^2\Vert u_k^\perp \Vert ^2 \\&=\mathfrak {d}_k(t)^2(1-\Vert u_k^\perp \Vert ^2) + \sum _{l=k-1}^{1} (\mathfrak {d}_l(t)^2-\mathfrak {d}_k(t)^2) |\langle u_k,\phi _l\rangle |^2 +\delta _k(t)^2\Vert u_k^\perp \Vert ^2. \end{aligned}$$

The conclusion (17) up to $k-1$, implies $|\langle u_l,\phi _l\rangle |^2\ge 1-\varepsilon _l^2$ for $l=1,\ldots ,k-1$. Since $\langle u_k,u_l\rangle =0$ for $l\not =k$,

$$\begin{aligned} |\langle u_l,\phi _l\rangle | |\langle u_k,\phi _l\rangle |= |\langle u_k,u_l-\langle u_l,\phi _l\rangle \phi _l\rangle |. \end{aligned}$$

Then, the Cauchy–Schwarz inequality alongside with (17) yield

21 $$\begin{aligned} |\langle u_k,\phi _l\rangle |^2 \le \frac{\varepsilon _l^2}{1-\varepsilon _l^2}. \end{aligned}$$

Hence, since $$\mathfrak {d}_l(t)\le \mathfrak {d}_k(t)$$,

$$\begin{aligned} F_k(t)^2 \ge \mathfrak {d}_{{k}}(t)^2 + \sum _{l=k-1}^{1} (\mathfrak {d}_l(t)^2-\mathfrak {d}_k(t)^2) \frac{\varepsilon _l^2}{1-\varepsilon _l^2} +(\delta _k(t)^2-\mathfrak {d}_k(t)^2)\Vert u_k^\perp \Vert ^2. \end{aligned}$$

Clearing $\Vert u_k^\perp \Vert ^2$ from this inequality and combining with the validity of (21) and (17) up to $k-1$, yields $\Pi _{\mathcal {S}_k}u_k\not =0$.

Let $\phi _k$ be as in (20). Then (17) is guaranteed for $j=k$. On the other hand, (17) up to $j=k$, (21) and the identity

$$\begin{aligned} F_k(t)^2 = \mathfrak {d}_k(t)^2|\langle u_k,\phi _k\rangle |^2 + \Vert (A-t) (u_k-\langle u_k,\phi _k\rangle \phi _k)\Vert ^2, \end{aligned}$$

yield (18) up to $j=k$. $\square$

#### Remark 2

If $t=\frac{\mathfrak {n}_j^-(t)+\mathfrak {n}_j^+(t)}{2}$ for a given *j*, the vectors $\phi _j^t$ introduced in Proposition 7 (and invoked subsequently) might not be eigenvectors of *A* despite the fact that $|A-t|\phi _j^t=\mathfrak {d}_j(t) \phi _j^t$. However, in any other circumstance $\phi _j^t$ are eigenvectors of *A*.

## Local bounds for eigenvalues

Our next purpose is to characterise the optimal parameters $t^\pm$ in Proposition 2 (Remark 1) by means of the following weak eigenvalue problem,

22 $$\begin{aligned}&\text {find } u\in \mathcal {L}{\setminus } \{0\}\quad \text { and }\; \tau \in \mathbb R\; \text { such that}\nonumber \\&\tau q_t(u, v) = l_t( u,v) \quad \forall v\in \mathcal {L}. \end{aligned}$$

This problem is central to the method for calculating eigenvalue bounds considered by Zimmermann and Mertins in [35]. Note that Assumption 1 ensures that (22) is well-posed.

Let

$$\begin{aligned} \tau ^-_{1}(t)\le \cdots \le \tau ^-_{n^-}(t)<0 \quad \text { and } \quad 0<\tau ^+_{n^+}(t)\le \cdots \le \tau ^+_{1}(t), \end{aligned}$$

be the negative and positive eigenvalues of (22), respectively. Here and below $n^\mp (t)$ are the number of these negative and positive eigenvalues, respectively. Both these quantities are piecewise constant in *t*. Below we will denote eigenfunctions associated with $\tau ^\mp _{j}(t)$ by $u_j^\mp (t)$.

Below we write most statements only for the case of “lower bounds for the eigenvalues of *A* which are to the left of *t*”. As the position of *t* relative to the essential spectrum is irrelevant here, evidently this does not restrict generality. The corresponding results regarding “upper bounds for the eigenvalues of *A* which are to the right of *t*” can be recovered by replacing *A* by $-A$.

The left side of (14) ensures the existence of $\tau ^-_1(t)$.

### Lemma 8

The following conditions are equivalent,

(a$$^-$$)
$$F_1(s)>t-s$$ for all $$s<t$$
(b$$^-$$)
$$\frac{\langle Au,u\rangle }{\langle u,u\rangle }>t$$ for all $$u\in \mathcal {L}$$
(c$$^-$$)
all the eigenvalues of (22) are positive.

### Remark 3

Let $\mathcal {L}={\text {Span}}\{b_j\}_{j=1}^n$. The matrix $[q_t(b_j,b_k)]_{jk=1}^n$ is singular if and only if $\mathcal {E}_t(A)\cap \mathcal {L}\ne \{0\}$. On the other hand, the kernel of (22) might be non-empty. If $n_0(t)$ is the dimension of this kernel and $n_\infty (t)=\dim (\mathcal {E}_t(A)\cap \mathcal {L})$, then $n=n_\infty (t)+n_0(t)+n^-(t)+n^+(t)$.

Note that $n_\infty (t)\ge 1$ if and only if $F_j(t)=0$ for $j=1,\ldots ,n_{\infty }(t)$. In this case the conclusions of Lemma 9 and Theorem 10 below do not have any meaning. In order to write our statements in a more transparent fashion we use Assumption 1.

By virtue of the next three statements, finding the negative eigenvalues of (22) is equivalent to finding $s=\hat{t}^-_j \in \mathbb R$ such that

23 $$\begin{aligned} t-s= F_j(s), \end{aligned}$$

and in this case $\hat{t}^{-}_j=t+\frac{1}{2\tau _j^-(t)}$. It then follows from Remark 1 that (22) encodes information about the optimal bounds for the spectrum around *t*, achievable by (13) in Proposition 2.

### The eigenvalue to the immediate left of *t*

We begin with the case $j=1$, see [23, Theorem 11].

#### Lemma 9

Let $t\in \mathbb R$ and $\mathcal {L}$ satisfy Assumption 1. The smallest eigenvalue $\tau =\tau _1^-(t)$ of (22) is negative if and only if there exists $s<t$ such that (23) holds true. In this case $s=t+\frac{1}{2\tau ^-_1(t)}$ and

$$\begin{aligned} F_1( s )=-\frac{1}{2\tau ^-_1(t)}= \frac{|u_1^-(t)|_s}{\Vert u_1^-(t)\Vert } \end{aligned}$$

for $u=u^-_1(t)\in \mathcal {L}$ the corresponding eigenvector.

#### Proof

For all $u\in \mathcal {L}$ and $s\in \mathbb R$,

$$\begin{aligned} q_s(u,u) - F_1(s)^2\langle u,u \rangle = q_t(u,u) + 2(t-s)l_t(u,u) +\left( (t-s)^2-F_1(s)^2\right) \langle u,u \rangle . \end{aligned}$$

Suppose that $F_1(s)=t-s$. Then

$$\begin{aligned} q_s(u,u) - F_1(s)^2\langle u,u \rangle = q_t(u,u) +2F_1(s)l_t(u,u). \end{aligned}$$

As the left side of this expression is non-negative,

$$\begin{aligned} \frac{l_t(u,u)}{q_t(u,u)}\ge -\frac{1}{2F_1(s)} \end{aligned}$$

for all $u\in \mathcal {L}{\setminus } \{0\}$ and the equality holds for some $u\in \mathcal {L}$. Hence $-\frac{1}{2F_1(s)}$ is the smallest eigenvalue of (22), and thus necessarily equal to $\tau _1^-(t)$. In this case $s-F_1(s)=t-2F_1(s)=t+\frac{1}{\tau _1^-(t)}$. Here the vector *u* for which equality is achieved is exactly $u=u^-_1(t)$.

Conversely, let $\tau ^-_1(t)$ and $u^-_1(t)$ be as stated. Then

$$\begin{aligned} \tau ^-_1(t)\le \frac{l_t(u,u)}{q_t(u,u)} \end{aligned}$$

for all $u\in \mathcal {L}$ with equality for $u=u^-_1(t)$. Re-arranging this expression yields

$$\begin{aligned} q_t(u,u) - \frac{1}{\tau ^-_1(t)} l_t(u,u) \ge 0 \end{aligned}$$

for all $u\in \mathcal {L}$ with equality for $u=u^-_1(t)$. The substitution $t=s-\frac{1}{2\tau ^-_1(t)}$ then yields

$$\begin{aligned} q_t(u,u) -\frac{1}{(2\tau _1^-(t))^2} \langle u,u\rangle \ge 0 \end{aligned}$$

for all $u\in \mathcal {L}$. The equality holds for $u=u^-_1(t)$. This expression can be further re-arranged as

$$\begin{aligned} \frac{|u|_s^2}{\Vert u\Vert ^2}\ge \frac{1}{(2\tau ^-_1(t))^2}. \end{aligned}$$

Hence $F_1(s)^2=\frac{1}{(2\tau _1^-(t))^2}$, as needed. $\square$

### Subsequent eigenvalues

An extension of Lemma 9 to the case $j\not =1$ is now found by induction.

#### Theorem 10

Let $1\le j\le n$ be fixed. The number of negative eigenvalues $n^-(t)$ of (22) is greater than or equal to *j* if and only if

$$\begin{aligned} \frac{\langle Au,u\rangle }{\langle u,u \rangle }< t \quad \text {for some} \quad u\in \mathcal {L}\ominus {\text {Span}}\{u^-_1(t),\ldots ,u^-_{j-1}(t) \}. \end{aligned}$$

Assuming this holds true, then $\tau =\tau ^-_j(t)$ and $u=u_j^-(t)$ are solutions of (22) if and only if

$$\begin{aligned} F_j\left( t+\frac{1}{2\tau _j^-(t)} \right) =- \frac{1}{2\tau _j^-(t)} = \frac{\left| u^-_j(t)\right| _{t+\frac{1}{2\tau _j^-(t)}}}{\Vert u^-_j(t)\Vert }. \end{aligned}$$

#### Proof

Recall that $t\in \mathbb R$ and $\mathcal {L}$ satisfy Assumption 1. For $j=1$ the statements are Lemma 9 taking into consideration (14). For $j>1$, due to the self-adjointness of the eigenproblem (22), it is enough to apply again Lemma 9 by fixing $\tilde{\mathcal {L}}=\mathcal {L}\ominus {\text {Span}}\{u^-_1(t),\ldots ,u^-_{j-1}(t) \}$ as trial spaces. Note that the negative eigenvalues of (22) for the trial space $\tilde{\mathcal {L}}$ are those of (22) for $\mathcal {L}$ except for $\tau _1^-(t),\ldots ,\tau _{j-1}^-(t)$. $\square$

A neat procedure for finding spectral bounds for *A*, as described in [35], can now be deduced from Theorem 10. By virtue of Proposition 2 and Remark 1, this procedure is optimal in the context of the approximated counting functions discussed in Sect. 3, see [23, Section 6]. We summarise the core statement as follows.

#### Corollary 11

For all $t\in \mathbb R$ and $j\in \{1,\ldots , n^\pm (t)\}$,

24 $$\begin{aligned} t+\frac{1}{\tau _j^-(t)}\le \mathfrak {n}_j^-(t) \quad \text {and} \quad \mathfrak {n}_j^+(t) \le t+\frac{1}{\tau _j^+(t)}. \end{aligned}$$

This corollary is an extension of the case $j=1$ established in [23, Theorem 11]. In recent years, numerical techniques based on this statement (for $j=1$) have been developed to successfully compute eigenvalues for the radially reduced magnetohydrodynamics operator [15, 35], the Helmholtz equation [6] and the calculation of sloshing frequencies [5]. We show an implementation to the case of the Maxwell operator with $j\ge 1$ in Sect. 6. See also [3].

## Convergence and error estimates

Our first goal in this section will be to show that, if $\mathcal {L}$ captures an eigenspace of *A* within a certain order of precision $\mathcal {O}(\varepsilon )$ as specified below, then the residuals

$$\begin{aligned} |t^\mp \mp F_j(t^\mp )-\mathfrak {n}_j^\mp (t) | \end{aligned}$$

(see the right side of (12)) are

- $$\mathcal {O}(\varepsilon )$$ for any $$t\in \mathbb R$$,
- $$\mathcal {O}(\varepsilon ^2)$$ for $$t\not \in \sigma (A)$$.

This will be the content of Theorems 13 and 14, and Corollary 15. We will then show that, in turns, (24) has always residual of order $\mathcal {O}(\varepsilon ^2)$ for any $t\in \mathbb R$. See Theorem 16. In the spectral approximation literature this property is known as optimal order of convergence/exactness, see [18, Chapter 6] or [33].

Recall Remark 2, and the Assumptions 1 and 2. Below $\{\phi _j^t\}_{j=1}^m$ denotes an orthonormal set of eigenvectors of $\mathcal {E}_{[t-\mathfrak {d}_m(t),t+\mathfrak {d}_m(t)]}(A)$ which is ordered so that

$$\begin{aligned} |A-t|\phi _j^t=\mathfrak {d}_j(t) \phi _j^t \quad \text {for} \quad j=1,\ldots , m. \end{aligned}$$

Whenever $0<\varepsilon _j<1$ is small, as specified below, the trial subspace $\mathcal {L}$ will be close to ${\text {Span}}\{\phi _j^t\}_{j=1}^m$ in the sense that there exist $w_j^t\in \mathcal {L}$ such that

$$\begin{aligned} \Vert w_j^t-\phi _j^t\Vert&\le \varepsilon _j \qquad \text {and} \qquad \qquad \qquad \qquad \qquad \, (\mathrm{A}_\mathrm{0})\\ |w_j^t-\phi _j^t|_t&\le \varepsilon _j. \qquad \qquad \qquad \qquad \qquad \qquad \qquad (\mathrm{A}_\mathrm{1}) \end{aligned}$$

We have split this condition into two terms, in order to highlight the fact that some times only (A$_\mathrm{1}$) is required. Unless otherwise specified, the index *j* runs from 1 to *m*. From Assumption 2 it follows that the family $\{\phi _j^s\}_{j=1}^m\subset \mathcal {E}_{[t-\mathfrak {d}_m(t),t+\mathfrak {d}_m(t)]}(A)$ and the family $\{w_j^s\}_{j=1}^m\subset \mathcal {L}$ above can always be chosen piecewise constant for *s* in a neighbourhood of *t*. Moreover, they can be chosen so that jumps only occur at $s\in \sigma (A)$.

A set $\{w_j^t\}_{j=1}^m$ subject to (A$_\mathrm{0}$)–(A$_\mathrm{1}$) is not generally orthonormal. However, according to the next lemma, it can always be substituted by an orthonormal set, provided $\varepsilon _j$ is small enough.

### Lemma 12

There exists $C>0$ independent of $\mathcal {L}$ ensuring the following. If $\{w_j^t\}_{j=1}^m\subset \mathcal {L}$ is such that (A$_\mathrm{0}$)-(A$_\mathrm{1}$) hold true for all $\varepsilon _j$ such that

$$\begin{aligned} \varepsilon =\sqrt{\sum _{j=1}^m\varepsilon _j^2}<\frac{1}{\sqrt{m}}, \end{aligned}$$

then there is a set $\{v_j^t\}_{j=1}^m\subset \mathcal {L}$ orthonormal in the inner product $\langle \cdot ,\cdot \rangle$ such that

$$\begin{aligned} |v_j^t-\phi _j^t|_t + \Vert v_j^t-\phi _j^t\Vert < C \varepsilon . \end{aligned}$$

Moreover, all these vectors are locally constant in *t* with jumps only at the spectrum of *A*.

### Proof

Recall Assumption 2. As it is clear from the context, in this proof we suppress the index *t* on top of any vector. The desired conclusion is achieved by applying the Gram–Schmidt procedure. Let $G=[\langle w_k,w_l\rangle ]_{kl=1}^m \in \mathbb {C}^{m\times m}$ be the Gram matrix associated to $\{w_j\}$. Set

$$\begin{aligned} v_j =\sum _{k=1}^m(G^{-1/2})_{kj}\;w_k. \end{aligned}$$

Then

$$\begin{aligned} \Vert G-I\Vert&\le \sqrt{\sum _{k,l=1}^m |\langle w_k,w_l\rangle -\langle \phi _k,\phi _l\rangle |^2}\\&\le \sqrt{2\sum _{k,l=1}^m \Vert w_k-\phi _k\Vert ^2(\Vert w_l\Vert +\Vert \phi _l\Vert )^2}\\&\le \sqrt{2}(2+\varepsilon )\varepsilon . \end{aligned}$$

Since

$$\begin{aligned} \Vert v_j-w_j\Vert ^2&= \left\| \sum _{k=1}^m(G^{-1/2}-I)_{kj}\;w_k\right\| ^2\\&= \sum _{k,l=1}^m(G^{-1/2}-I)_{kj} \overline{(G^{-1/2}-I)_{lj}}\langle w_k, w_{l} \rangle \\&= \sum _{k=1}^m (G^{-1/2}-I)_{kj} \overline{\left( \sum _{l=1}^m G_{kl}(G^{-1/2}-I)_{lj}\right) }\\&= \sum _{k=1}^m (G^{-1/2}-I)_{kj}(G^{1/2}-G)_{jk}\\&= \left( (I-G^{1/2})^2\right) _{jj} \end{aligned}$$

then

$$\begin{aligned} \Vert v_j-w_j\Vert \le \Vert I-G^{1/2}\Vert . \end{aligned}$$

As $G^{1/2}$ is a positive-definite matrix, for every $\underline{v}\in \mathbb {C}^m$ we have

$$\begin{aligned} \Vert (G^{1/2}+I)\underline{v}\Vert ^2=\Vert G^{1/2}\underline{v}\Vert ^2+2\langle G^{1/2}\underline{v},\underline{v}\rangle +\Vert \underline{v}\Vert ^2 \ge \Vert \underline{v}\Vert ^2. \end{aligned}$$

Then $\det (I+G^{1/2})\not =0$ and $\Vert (I+G^{1/2})^{-1}\Vert \le 1$. Hence

25 $$\begin{aligned} \Vert v_j-w_j\Vert \le \Vert (I-G)(I+G^{1/2})^{-1}\Vert \le \Vert I-G\Vert \,\Vert (I+G^{1/2})^{-1}\Vert \le (2+\varepsilon )\varepsilon . \end{aligned}$$

Now, identify $\underline{v}=(v_1,\ldots ,v_m)\in {\mathbb C}^m$ with $v=\sum _{k=1}^m v_k\phi _k$. As

$$\begin{aligned} \Vert G^{1/2} \underline{v}\Vert =\left\| \sum _{j=1}^m \langle v, \phi _j\rangle w_j \right\| \ge \Vert v\Vert - \left\| \sum _{j=1}^m \langle v, \phi _j\rangle (w_j-\phi _j)\right\| \ge (1-\varepsilon )\Vert \underline{v}\Vert \end{aligned}$$

then

$$\begin{aligned} \Vert G^{-1/2}\Vert \le \frac{1}{1-\varepsilon }. \end{aligned}$$

Hence

26 $$\begin{aligned} |v_j-w_j|_t&\le \sum _{k=1}^m|(G^{-1/2}-I)_{jk}||w_k|_t \nonumber \\&\le \sum _{k=1}^m|(G^{-1/2}-I)_{jk}|(\varepsilon _k+\mathfrak {d}_k(t)) \nonumber \\&\le \sum _{k,l=1}^m|(G^{-1/2})_{kl}||(G^{1/2}-I)_{lj}|(\varepsilon _k+\mathfrak {d}_k(t)) \nonumber \\&\le \frac{\sqrt{m}(\varepsilon +\mathfrak {d}_m(t)) (2+\varepsilon )}{1-\varepsilon }\varepsilon . \end{aligned}$$

The conclusion follows from (25) and (26). $\square$

### Convergence of the approximated local counting function

The next theorem addresses the claim (a) made at the beginning of this section. According to Lemma 12, in order to examine the asymptotic behaviour of $F_j(t)$ as $\varepsilon _j\rightarrow 0$ under the constraints (A$_\mathrm{0}$)–(A$_\mathrm{1}$), without loss of generality the trial vectors $w_j^t$ can be assumed to form an orthonormal set in the inner product $\langle \cdot ,\cdot \rangle$.

#### Theorem 13

Let $\{w_j^t\}_{j=1}^m\subset \mathcal {L}$ be a family of vectors which is orthonormal in the inner product $\langle \cdot ,\cdot \rangle$ and satisfies (A$_\mathrm{1}$). Then

$$\begin{aligned} F_j(t)-\mathfrak {d}_j(t) \le \left( \sum _{k=1}^j \varepsilon _k^2 \right) ^{1/2}\quad \forall j=1,\ldots ,m. \end{aligned}$$

#### Proof

Recall Assumption 2. From the Rayleigh–Ritz principle we obtain

$$\begin{aligned} F_j(t)&\le \max _{\sum |c_k|^2=1} \left| \sum _{k=1}^j c_k w_k \right| _t \\&\le \max _{\sum |c_k|^2=1} \left| \sum _{k=1}^j c_k (w_k-\phi _k) \right| _t+ \max _{\sum |c_k|^2=1} \left| \sum _{k=1}^j c_k \phi _k\right| _t \\&= \max _{\sum |c_k|^2=1} \left| \sum _{k=1}^j c_k (w_k-\phi _k)\right| _t+\mathfrak {d}_j(t). \end{aligned}$$

This gives

$$\begin{aligned} F_j(t)-\mathfrak {d}_j(t)&\le \max _{\sum |c_k|^2=1} \sum _{k=1}^j |c_k| |w_k-\phi _k|_t \\&\le \max _{\sum |c_k|^2=1} \left( \sum _{k=1}^j |c_k|^2\right) ^{1/2} \left( \sum _{k=1}^j |w_k-\phi _k|_t^2 \right) ^{1/2} \le \left( \sum _{k=1}^j \varepsilon _k^2 \right) ^{1/2} \end{aligned}$$

as needed. $$\square $$

In terms of order of approximation, Theorem 13 will be superseded by Theorem 14 for $t\not \in \sigma (A)$. However, if $t\in \sigma (A)$, the trial space $\mathcal {L}$ can be chosen so that $F_1(t)-\mathfrak {d}_1(t)$ is only linear in $\varepsilon _1$. Indeed, fixing any non-zero $u\in {\text {D}}(A)$ and $\mathcal {L}={\text {Span}}\{u\}$, yields $F_1(t)-\mathfrak {d}_1(t)=F_1(t)=\varepsilon _1$. Therefore Theorem 13 is optimal, on the presumption that *t* is arbitrary.

The next theorem addresses the claim (b) made at the beginning of this section. Its proof is reminiscent of that of [29, Theorem 6.1].

#### Theorem 14

Let $t\not \in \sigma (A)$. Suppose that the $\varepsilon _j$ in (A$_\mathrm{1}$) are such that

27 $$\begin{aligned} \sum _{j=1}^m \varepsilon _j^2<\frac{\mathfrak {d}_1(t)^2}{6}. \end{aligned}$$

Then,

28 $$\begin{aligned} F_j(t)-\mathfrak {d}_j(t)\le 3\frac{\mathfrak {d}_j(t)}{\mathfrak {d}_1(t)^2 } \sum _{k=1}^j \varepsilon _k^2 \quad \forall j=1,\ldots ,m. \end{aligned}$$

#### Proof

Recall Assumption 2. Since $t\not \in \sigma (A)$, then $({\text {D}}(A),q_t(\cdot ,\cdot ))$ is a Hilbert space. Let $P_\mathcal {L}:{\text {D}}(A)\longrightarrow \mathcal {L}$ be the orthogonal projection onto $\mathcal {L}$ with respect to the inner product $q_t(\cdot ,\cdot )$, so that

$$\begin{aligned} q_t(u-P_{\mathcal {L}}u,v)=0 \quad \forall v\in \mathcal {L}. \end{aligned}$$

Then $|u|_t^2=|P_\mathcal {L}u|_t^2+|u-P_\mathcal {L}u|_t^2$ for all $u\in {\text {D}}(A)$ and $|u-P_{\mathcal {L}}u|_t \le |u-v|_t$ for all $v\in \mathcal {L}$. Hence

29 $$\begin{aligned} |\phi _k-P_{\mathcal {L}} \phi _k|_t \le \varepsilon _k \quad \forall k=1,\ldots ,m. \end{aligned}$$

Let $$\mathcal {E}_j=\mathrm {Span}\{\phi _k\}_{k=1}^j$$. Define

$$\begin{aligned} \mathcal {F}_j&=\{\phi \in \mathcal {E}_j : \Vert \phi \Vert =1 \} \quad \text {and} \\ \mu _{\mathcal {L}}^j(t)&=\max _{\phi \in \mathcal {F}_j} \left| 2 {\text {Re}}\langle \phi , \phi -P_{\mathcal {L}} \phi \rangle -\Vert \phi -P_{\mathcal {L}}\phi \Vert ^2 \right| . \end{aligned}$$

Here $\mu _{\mathcal {L}}^j$ depends on *t*, as $P_\mathcal {L}$ does. We first show that, under hypothesis (27), $\mu _\mathcal {L}^j(t)<\frac{1}{2}$. Indeed, given $\phi \in \mathcal {F}_j$ we decompose it as $\phi =\sum _{k=1}^j c_k \phi _k$. Then

30 $$\begin{aligned} |\langle \phi ,\phi -P_\mathcal {L}\phi \rangle |&= \left| \sum _{k=1}^j c_k \langle \phi _k, \phi -P_\mathcal {L}\phi \rangle \right| =\left| \sum _{k=1}^j \frac{c_k}{\mathfrak {d}_k(t)^2} q_t(\phi _k, \phi -P_\mathcal {L}\phi ) \right| \nonumber \\&=\left| q_t\left( \sum _{k=1}^j \frac{c_k}{\mathfrak {d}_k(t)^2}\phi _k, \phi -P_\mathcal {L}\phi \right) \right| \nonumber \\&= \left| q_t\left( \sum _{k=1}^j \frac{c_k}{\mathfrak {d}_k(t)^2}(\phi _k-P_\mathcal {L}\phi _k), \phi -P_\mathcal {L}\phi \right) \right| \nonumber \\&\le \left| \sum _{k=1}^j \frac{c_k}{\mathfrak {d}_k(t)^2}(\phi _k-P_\mathcal {L}\phi _k) \right| _t\, \left| \sum _{k=1}^j c_k(\phi _k-P_\mathcal {L}\phi _k) \right| _t. \end{aligned}$$

For each multiplying term in the latter expression, the triangle and Cauchy–Schwarz’s inequalities yield (take $\alpha _k=c_k$ or $\alpha _k=\frac{c_k}{\mathfrak {d}_k(t)^2}$)

31 $$\begin{aligned} \left| \sum _{k=1}^j \alpha _k (\phi _k-P_\mathcal {L}\phi _k) \right| _t&\le \sum _{k=1}^j |\alpha _k|\, |\phi _k-P_\mathcal {L}\phi _k|_t \nonumber \\&\le \left( \sum _{k=1}^j |\alpha _k|^2\right) ^{1/2} \left( \sum _{k=1}^j |\phi _k-P_\mathcal {L}\phi _k|_t^2 \right) ^{1/2}. \end{aligned}$$

Then

32 $$\begin{aligned} \left| 2{\text {Re}}\langle \phi ,\phi -P_\mathcal {L}\phi \rangle \right|\le & {} 2\left( \sum _{k=1}^j \frac{|c_k|^2}{\mathfrak {d}_k(t)^4}\right) ^{1/2} \left( \sum _{k=1}^j |c_k|^2\right) ^{1/2} \sum _{k=1}^j \varepsilon _k^2 \nonumber \\\le & {} \frac{2}{\mathfrak {d}_1(t)^2}\sum _{k=1}^j \varepsilon _k^2 \end{aligned}$$

for all $$\phi \in \mathcal {F}_j$$.

The other term in the expression for $\mu _\mathcal {L}^j(t)$ has an upper bound found as follows. According to the Rayleigh–Ritz principle

33 $$\begin{aligned} \Vert \phi -P_\mathcal {L}\phi \Vert ^2 \le \frac{1}{\mathfrak {d}_1(t)^2}q_t(\phi -P_\mathcal {L}\phi ,\phi -P_\mathcal {L}\phi ). \end{aligned}$$

Therefore, by repeating analogous steps as in (30) and (31), we get

34 $$\begin{aligned} \Vert \phi -P_\mathcal {L}\phi \Vert ^2&\le \frac{1}{\mathfrak {d}_1(t)^2}\sum _{k=1}^jc_k q_t(\phi _k-P_\mathcal {L}\phi _k,\phi -P_\mathcal {L}\phi ) \nonumber \\&= q_t\left( \sum _{k=1}^j \frac{c_k}{\mathfrak {d}_1(t)^2}(\phi _k-P_\mathcal {L}\phi _k),\phi -P_\mathcal {L}\phi \right) \nonumber \\&= q_t\left( \sum _{k=1}^j \frac{c_k}{\mathfrak {d}_1(t)^2}(\phi _k-P_\mathcal {L}\phi _k),\sum _{l=1} ^jc_l(\phi _l-P_\mathcal {L}\phi _l)\right) \nonumber \\&\le \frac{1}{\mathfrak {d}_1(t)^2}\sum _{k=1}^j \varepsilon _k^2. \end{aligned}$$

Hence, from (32) and (34),

35 $$\begin{aligned} \mu _\mathcal {L}^j(t)\le \frac{3}{\mathfrak {d}_1(t)^2}\sum _{k=1}^j\varepsilon _k^2 < \frac{1}{2} \end{aligned}$$

as a consequence of (27).

Next, observe that $\dim (P_\mathcal {L}\mathcal {E}_j) = j$. Indeed $P_\mathcal {L}\psi =0$ for $\Vert \psi \Vert =1$ would imply

$$\begin{aligned} \mu _\mathcal {L}^j(t)\ge \left| 2 {\text {Re}}\langle \psi , \psi -P_{\mathcal {L}} \psi \rangle -\Vert \psi -P_{\mathcal {L}}\psi \Vert ^2 \right| =\Vert \psi \Vert ^2=1, \end{aligned}$$

which would contradict the fact that $\mu _\mathcal {L}^j(t)<1$. Then,

$$\begin{aligned} F_j (t)^2\le \max _{u\in P_\mathcal {L}\mathcal {E}_j}\frac{|u|_t^2}{\Vert u\Vert ^2} =\max _{\phi \in \mathcal {E}_j}\frac{|P_\mathcal {L}\phi |_t^2}{\Vert P_\mathcal {L}\phi \Vert ^2} =\max _{\phi \in \mathcal {F}_j}\frac{|P_\mathcal {L}\phi |_t^2}{\Vert P_\mathcal {L}\phi \Vert ^2}. \end{aligned}$$

As

$$\begin{aligned} \Vert P_\mathcal {L}\phi \Vert ^2=\Vert \phi \Vert ^2-2{\text {Re}}\langle \phi ,\phi -P_\mathcal {L}\phi \rangle +\Vert \phi -P_\mathcal {L}\phi \Vert ^2 \ge 1-\mu ^j_\mathcal {L}(t), \end{aligned}$$

we get

36 $$\begin{aligned} F_j(t)^2\le \max _{\phi \in \mathcal {F}_j} \frac{|\phi |_t^2}{1-\mu _\mathcal {L}^j(t)}= \max _{\sum |c_k|^2=1} \frac{\sum _{k=1}^j |c_k|^2 \mathfrak {d}_k(t)^2}{1-\mu _\mathcal {L}^j(t)}=\frac{\mathfrak {d}_j(t)^2}{1-\mu _\mathcal {L}^j(t)}. \end{aligned}$$

Finally, (36) and (35) yield

37 $$\begin{aligned} F_j(t)^2-\mathfrak {d}_j(t)^2&\le \frac{\mu _\mathcal {L}^j(t)}{1-\mu _\mathcal {L}^j(t)}\mathfrak {d}_j(t)^2 \nonumber \\&\le 2 \mu _\mathcal {L}^j(t)\mathfrak {d}_j(t)^2\nonumber \\&\le 2\frac{3}{\mathfrak {d}_1(t)^2}\mathfrak {d}_j(t)^2\sum _{k=1}^j\varepsilon _k^2. \end{aligned}$$

The proof is completed by observing that $F_j(t)+\mathfrak {d}_j(t)\ge 2\mathfrak {d}_j(t).$ $\square$

As the next corollary shows, a quadratic order of decrease for $F_j(t)-\mathfrak {d}_j(t)$ is prevented for $t\in \sigma (A)$ (in the context of Theorems 13 and 14), only for *j* up to $\dim \mathcal {E}_{t}(A)$.

#### Corollary 15

Let $t\in \sigma _{\mathrm {disc}}(A)$, $\ell =1+\dim \mathcal {E}_{t}(A)$ and $k\in \{\ell ,\ldots ,m\}$. Let

$$\begin{aligned} \alpha _k(t)= \frac{1}{4} \min \left\{ |\mathfrak {d}_l(t)-\mathfrak {d}_{l-1}(t)|: \mathfrak {d}_l(t)\not = \mathfrak {d}_{l-1}(t), \, l=\ell ,\ldots ,k\right\} >0. \end{aligned}$$

There exists $\varepsilon >0$ independent of *k* ensuring the following. If (A$_\mathrm{1}$) holds true for $\sqrt{\sum _{j=1}^m \varepsilon _j^2}<\varepsilon$, then

$$\begin{aligned} F_k(t)-\mathfrak {d}_k(t)\le 3\frac{\mathfrak {d}_k(t)}{\alpha _k(t)^2} \sum _{j=1}^k \varepsilon _j^2. \end{aligned}$$

#### Proof

Without loss of generality we assume that $t+\mathfrak {d}_k(t)\in \sigma (A)$. Otherwise $t-\mathfrak {d}_k(t)\in \sigma (A)$ and the proof is analogous to the one presented below.

Let $\tilde{t}=t+\alpha _k(t)$. Then $\tilde{t}\not \in \sigma (A)$ and $t+\mathfrak {d}_k(t)=\tilde{t}+\mathfrak {d}_k(\tilde{t})$. Since the map $s\mapsto s+F_j(s)$ is non-decreasing as a consequence of Proposition 2, Theorem 14 applied at $\tilde{t}$ yields

$$\begin{aligned} F_k(t)-\mathfrak {d}_k(t)&= t+F_k(t)-(t+\mathfrak {d}_k(t)) \le \tilde{t}+F_k(\tilde{t})-(\tilde{t}+\mathfrak {d}_k(\tilde{t}))\\&= F_k(\tilde{t})-\mathfrak {d}_k(\tilde{t}) \le 3\frac{\mathfrak {d}_k(\tilde{t})}{\mathfrak {d}_1(\tilde{t})^2}\sum _{j=1}^k\varepsilon _k^2\le 3 \frac{\mathfrak {d}_k(t)}{\alpha _k(t)^2}\sum _{j=1}^k\varepsilon _j^2 \end{aligned}$$

as needed. $$\square $$

### Convergence of local bounds for eigenvalues

Our next task in this section is to formulate precise statements on the convergence of the method of Zimmermann and Mertins (Sect. 4). Theorem 16 below improves upon two crucial aspects of a similar result established in [15, Lemma 2]. It allows $j>1$ and it allows $t\in \sigma (A)$. These two improvements are essential in order to obtain sharp bounds for those eigenvalues which are either degenerate or form a tight cluster.

#### Remark 4

The constants $\tilde{\varepsilon }_t$ and $C_t^\pm$ below do have a dependence on *t*. This dependence can be determined explicitly from Theorem 14, Corollary 15 and the proof of Theorem 16. Despite the fact that these constants can deteriorate as *t* approaches the isolated eigenvalues of *A* and they can have jumps precisely at these points, they may be chosen independent of *t* on compact sets outside the spectrum.

#### Remark 5

By virtue of Corollary 11 and Corollary 6, $\frac{1}{\tau _j^-(t)}\le \nu _j^-(t)-t$ and $\frac{1}{\tau _j^+(t)}\ge \nu _j^+(t)-t$. Then

$$\begin{aligned} \hat{t}^-_j=t+\frac{1}{2\tau _j^-(t)}\le \frac{t+\nu _j^-(t)}{2} \le \frac{\nu _j^+(t)+\nu _j^-(t)}{2} \le \frac{\nu _j^+(t)+t}{2}\le t+\frac{1}{2\tau _j^+(t)} =\hat{t}^+_j. \end{aligned}$$

We regard the following as one of the main results of this work.

#### Theorem 16

Let $J\subset \mathbb R$ be a bounded open segment such that $J\cap \sigma (A)\subseteq \sigma _\mathrm {disc}(A)$. Let $\{\phi _k\}_{k=1}^{\tilde{m}}$ be a family of eigenvectors of *A* such that ${\text {Span}}\{\phi _k\}_{k=1}^{\tilde{m}}=\mathcal {E}_J(A)$. For fixed $t\in J$ such that Assumption 1 is satisfied, there exist $\tilde{\varepsilon }_t>0$ and $C^{-}_t>0$ independent of the trial space $\mathcal {L}$, ensuring the following. If there are $\{w_j\}_{j=1}^{\tilde{m}}\subset \mathcal {L}$ such that

38 $$\begin{aligned} \left( \sum _{j=1}^{\tilde{m}} \Vert w_j-\phi _j\Vert ^2+|w_j-\phi _j|_t^2\right) ^{1/2} \le \varepsilon <\tilde{\varepsilon }_t, \end{aligned}$$

then

$$\begin{aligned} 0< \nu ^-_j(t)-\left( t+\frac{1}{\tau _j^-(t)}\right) \le C_t^- \varepsilon ^2 \end{aligned}$$

for all $j \le n^-(t)$ such that $\nu ^-_j(t)\in J$.

#### Proof

The hypotheses ensure that the number of indices $j\le n^-(t)$ such that $\nu ^-_j(t)\in J$ never exceeds $\tilde{m}$. Therefore this condition in the conclusion of the theorem is consistent.

Let

$$\begin{aligned} m(t)=\max \{m\in \mathbb {N}:[t-\mathfrak {d}_m(t),t+\mathfrak {d}_m(t)]\subset J\}. \end{aligned}$$

The hypothesis on $\mathcal {L}$ guarantees that (A$_\mathrm{0}$)–(A$_\mathrm{1}$) hold true for $m=m(t)$ and $(\sum _{j=1}^{m}\varepsilon _j^2)^{1/2} <\varepsilon$. By combining Lemma 12 and Theorem 13 and the fact that we can pick $\{w^t_j\}_{j=1}^{m(t)}\subseteq \{w_k\}_{k=1}^{\tilde{m}}$, there exists $\tilde{\varepsilon }_t>0$ small enough, such that (38) yields

39 $$\begin{aligned} F_j(s)-\mathfrak {d}_j(s)\le {\frac{t-\nu _1^-(t)}{2}} \qquad \forall j=1,\ldots ,\tilde{m}\quad \text {and} \quad s\in J. \end{aligned}$$

Let *j* be such that $\nu ^-_j(t)\in J$. Since $\nu _j^-(t)-(\alpha +t) \le (t+\alpha )- \nu _1^-(t)$ for all $\alpha$ such that $\frac{\nu _j^-(t)+\nu _1^-(t)}{2}-t\le \alpha \le 0$, then

$$\begin{aligned} \mathfrak {d}_j(s)=s-\nu _j^-(t) \qquad \forall s\in \left[ \frac{\nu _1^-(t)+\nu _j^-(t)}{2},\frac{t+\nu _j^-(t)}{2}\right] . \end{aligned}$$

Let

$$\begin{aligned} g(\alpha )= F_j(t+\alpha )+\alpha . \end{aligned}$$

Then *g* is an increasing function of $\alpha$ and $g(0)=F_j(t)>0$. For the strict inequality in the latter, recall Assumption 1. Moreover, according to (39),

$$\begin{aligned} g\left( \frac{\nu _j^-(t)+\nu _1^-(t)}{2}-t\right)&= F_j\left( \frac{\nu _j^-(t)+\nu _1^-(t)}{2}\right) -t+\nu _1^-(t) - \frac{\nu _1^-(t)-\nu _j^-(t)}{2} \\&= F_j\left( \frac{\nu _j^-(t)+\nu _1^-(t)}{2}\right) -t+\nu _1^-(t) - \mathfrak {d}_j\left( \frac{\nu _j^-(t)+\nu _1^-(t)}{2}\right) \\&\le \frac{t-\nu _1^-(t)}{2} -(t-\nu _1^-(t)) < 0. \end{aligned}$$

Hence, the intermediate value theorem ensures the existence of $\tilde{\alpha }\in (\frac{\nu _1^-(t)+\nu _j^-(t)}{2}-t,0)$ such that $\tilde{\alpha }=F_j(t+\tilde{\alpha })$. According to Theorem 10, $\tilde{\alpha }$ is unique and $\tilde{\alpha }=\frac{1}{2\tau _j^-(t)}$.

The proof is now completed as follows. By virtue of Remark 5,

$$\begin{aligned} \hat{t}_j^-(t)=t+\frac{1}{2\tau _j^-(t)}\in \left( \frac{\nu _1^-(t)+\nu _j^-(t)}{2}, \frac{t+\nu _j^-(t)}{2}\right) \quad \text {and} \quad F_j(\hat{t}_j^-(t))=\frac{1}{2\tau _j^-(t)}. \end{aligned}$$

Then, Theorem 14 or Corollary 15, as appropriate, ensure the existence of $C_t^->0$ yielding

$$\begin{aligned} \nu _j^-(t)-\left( t+\frac{1}{\tau _j^-(t)}\right) ={F_j}(\hat{t} _j^-)-\mathfrak {d}_j(\hat{t}^-_j)\le C_t^-\sum _{k=1}^j\varepsilon _k^2<C_t^- \varepsilon ^2, \end{aligned}$$

as needed. $$\square $$

### Convergence to eigenfunctions

We conclude this section with a statement on convergence to eigenfunctions.

#### Corollary 17

Let $J\subset \mathbb R$ be a bounded open segment such that $J\cap \sigma (A)\subseteq \sigma _\mathrm {disc}(A)$. Let $\{\phi _k\}_{k=1}^{\tilde{m}}$ be a family of eigenvectors of *A* such that ${\text {Span}}\{\phi _k\}_{k=1}^{\tilde{m}}=\mathcal {E}_J(A)$. For fixed $t\in J$, there exist $\tilde{\varepsilon }_t>0$ and $C^{\pm }_t>0$ independent of the trial space $\mathcal {L}$, ensuring the following. If there are $\{w_j\}_{j=1}^{\tilde{m}}\subset \mathcal {L}$ such that (38) holds, then for all $j\le n^\pm (t)$ such that $\nu _j^\pm (t)\in J$ we can find $\psi _j^{\varepsilon \pm }\in \mathcal {E}_{\{\nu _j^-(t),\nu _j^+(t)\}}(A)$ satisfying

$$\begin{aligned} |u_j^\pm (t)-\psi _j^{\varepsilon \pm }|_t+ \Vert u_j^\pm (t)-\psi _j^{\varepsilon \pm }\Vert \le C_t^\pm \varepsilon . \end{aligned}$$

#### Proof

Fix $t\in J$. According to Theorem 10, $u^\pm _j(t)=u^{\hat{t}^\pm _j}_j$ in the notation for eigenvectors employed in Proposition 7. The claimed conclusion is a consequence of the latter combined with Theorem 14 or Corollary 15, as appropriate. $\square$

#### Remark 6

Once again, we remark that the vectors in the statement of the corollary can be chosen locally constant in *t* with jumps only at the spectrum of *A*.

## Implementations to the Maxwell eigenvalue problem

The method of Zimmermann and Mertins can be applied to a large variety of self-adjoint operators. Of particular interest are the operators which are not bounded below or above. A significant class of block operator matrices [31] which are highly relevant in applications fall into this category and are covered by the present framework. In order to illustrate our findings in this setting, we now apply the method of Zimmermann and Mertins to the Maxwell operator. This operator has been extensively studied in the last few years with a special emphasis on the spectral pollution phenomenon.

Let $\Omega \subset \mathbb R^3$ be a polyhedron which is open, bounded, simply connected and Lipschitz in the sense of [1, Notation 2.1]. Let $\partial \Omega$ be the boundary of $\Omega$ and denote by $\mathbf {n}$ its outer normal vector. The physical phenomenon of electromagnetic oscillations in a resonator filled with a homogeneous medium is described by the isotropic Maxwell eigenvalue problem,

40 $$\begin{aligned} \left\{ \begin{array}{lll} &{} {\text {curl}}~{\varvec{E}}= i\omega {\varvec{H}}&{} \text{ in } \Omega \\ &{} {\text {curl}}~{\varvec{H}}= -i\omega {\varvec{E}}&{} \text {in }\Omega \\ &{} {\varvec{E}}\times \mathbf {n}=0&{} \text {on } \partial \Omega . \end{array} \right. \end{aligned}$$

Here the angular frequency $\omega \in \mathbb R$ and the field phasor $({\varvec{E}},{\varvec{H}}) \not =0$ is restricted to the solenoidal subspace, characterised by the Gauss law

41 $$\begin{aligned} {\text {div}}({\varvec{E}})=0={\text {div}}({\varvec{H}}), \end{aligned}$$

but when $\omega \ne 0$ note that the Gauss law is redundant in (40). See [10].

The orthogonal complement of this subspace is the gradient space, which has infinite dimension and lies in the kernel of the eigenvalue equation (40). Here, we use the term “kernel” to refer to the solution of the eigenvalue problem associated to $\omega =0$. In turns, this means that (40), (41) and the unrestricted problem (40), have the same non-zero spectrum and the same corresponding eigenspaces.

Let

$$\begin{aligned} \mathcal {H}({\text {curl}};\Omega )=\left\{ \varvec{u}\in [L^2(\Omega )]^3 : {\text {curl}}~\varvec{u}\in [L^2(\Omega )]^3 \right\} \end{aligned}$$

be equipped with the norm

42 $$\begin{aligned} \Vert \varvec{u}\Vert _{{\text {curl}},\Omega }^2=\Vert \varvec{u}\Vert _{0,\Omega }^2+\Vert {\text {curl}}~\varvec{u}\Vert _{0,\Omega }^2. \end{aligned}$$

Let $\mathcal {R}_{\max }$ denote the operator defined by the expression “${\text {curl}}~$” acting on the domain ${\text {D}}(\mathcal {R}_{\max })=\mathcal {H}({\text {curl}};\Omega )$, the maximal domain. Let

$$\begin{aligned} \mathcal {R}_{\min }=\mathcal {R}_{\max }^*=\overline{\mathcal {R}_{\max } \!\upharpoonright \! [\mathcal D(\Omega )]^3}. \end{aligned}$$

The domain of $\mathcal {R}_{\min }$ is

$$\begin{aligned} {\text {D}}(\mathcal {R}_{\min })&= \mathcal {H}_0({\text {curl}};\Omega ) \\&= \{ \varvec{u}\in \mathcal {H}({\text {curl}};\Omega ) :\langle {\text {curl}}~\varvec{u},\varvec{v}\rangle _\Omega = \langle \varvec{u},{\text {curl}}~\varvec{v}\rangle _\Omega \quad \forall \varvec{v}\in \mathcal {H}({\text {curl}};\Omega )\}. \end{aligned}$$

By virtue of Green’s identity for the rotational [25, Theorem I.2.11],

$$\begin{aligned} \mathcal {H}_0({\text {curl}};\Omega )=\{ \varvec{u}\in \mathcal {H}({\text {curl}};\Omega ): \varvec{u}\times \mathbf {n}={\mathbf 0} \;\mathrm {on}\;\partial \Omega \}. \end{aligned}$$

The linear operator associated to (40) is then,

$$\begin{aligned} \mathcal M= \begin{pmatrix} 0 &{} i \mathcal {R}_{\max } \\ -i \mathcal {R}_{\min } &{} 0 \end{pmatrix} \end{aligned}$$

on the domain

43 $$\begin{aligned} {\text {D}}(\mathcal M)={\text {D}}(\mathcal {R}_{\min })\times {\text {D}}(\mathcal {R}_{\max })\subset [L^2(\Omega )]^6. \end{aligned}$$

Note that $\mathcal M:{\text {D}}(\mathcal M)\longrightarrow [L^2(\Omega )]^6$ is self-adjoint, as $\mathcal {R}_{\max }$ and $\mathcal {R}_{\min }$ are mutually adjoints [10, Lemma 1.2].

The numerical estimation of the eigenfrequencies of (40)-(41) is known to be extremely challenging for general regions $\Omega$. The operator $\mathcal {M}$ does not have a compact resolvent and it is strongly indefinite. If we consider, instead, the problem (40)-(41), this would lead to a formulation involving an operator with a compact resolvent (due to (41)), but the problem would still be strongly indefinite. By considering the square of $\mathcal {M}$ on the solenoidal subspace, one obtains a positive definite eigenvalue problem (involving the bi-curl) which can be discretised via the Galerkin method. However, a serious drawback of this idea for practical computations is the fact that the standard finite element spaces are not solenoidal. Usually, spurious modes associated to the infinite-dimensional kernel appear and give rise to spectral pollution. This has been well documented and it is known to be a manifested problem when the underlying mesh is unstructured, see [2, 11] and references therein.

Various ingenious methods, e.g. [7, 11–14, 16, 17, 27], capable of approximating the eigenvalues of (40) by means of the finite element method have been documented in the past. In all the above-cited works, either a particular choice of finite element spaces, or an appropriate modification of the weak formulation of the problem, has to be performed prior to the computation of the eigenvalues.

The method of Zimmermann and Mertins does not need to introduce any prior change to the problem at hand in order to find eigenvalue bounds for $\mathcal {M}$. We can even pick $\mathcal {L}$ made of Lagrange finite elements on unstructured meshes. Convergence and absence of spectral pollution are guaranteed by Corollary 11 and Theorem 16. Our purpose below is only to illustrate the context of the theory presented in the previous sections. A more comprehensive numerical investigation of this model, including the case of anisotropic media, has been conducted in [3].

Let $\{\mathcal {T}_h\}_{h>0}$ be a family of shape-regular triangulations of $\overline{\Omega }$, [24], where each element $K\in {\mathcal {T}}_h$ is a simplex with diameter $h_K$ such that $h=\max _{K\in {\mathcal {T}}_h}h_K$. For $r\ge 1$, let

$$\begin{aligned} \mathbf {V}_h^r&=\{\varvec{v}_h\in [C^0(\overline{\Omega })]^3: \varvec{v}_h|_K \in [\mathbb {P}_r(K)]^3 \ \forall K\in \mathcal {T}_h \}, \\ \mathbf {V}_{h,0}^r&=\{\varvec{v}_h\in \mathbf {V}_h^r: \varvec{v}_h\times \mathbf {n}={\mathbf 0} \;\text {on}\;\partial \Omega \} \end{aligned}$$

and set

$$\begin{aligned} \mathcal {L}_h=\mathbf {V}_{h,0}^r\times \mathbf {V}_h^r \subset {\text {D}}(\mathcal M). \end{aligned}$$

Let $\omega _1\le \omega _2 \le \cdots$ be the positive eigenvalues of $\mathcal {M}$. The upper bounds $\omega _j^+$ and lower bounds $\omega ^-_j$ reported below are found by fixing $t\in \mathbb {R}$, solving (22) for $\mathcal {L}=\mathcal {L}_h$ numerically, and then applying (24).

The only hypothesis required in the analysis carried out in Sect. 5, ensuring that the $\omega ^\pm _j$ are close to $\omega _j$, is for the trial space to capture well the eigenfunctions in the graph norm of ${\text {D}}(\mathcal M)$, that is the $[\mathcal {H}({\text {curl}},\Omega )]^2$-norm. See (38). Therefore, as we have substantial freedom to choose these spaces and they constitute the simplest alternative, we have picked the Lagrange nodal elements.

A direct application of Theorem 16, Corollary 17, and classical interpolation estimates e.g. [19, Theorem 3.1.6], leads to convergence of the approximated eigenvalues and eigenspaces. To be precise, in [3, Theorem 3.3] the following results are proven. For all $j\in \mathbb {N}$,

$$\begin{aligned} \lim _{h\rightarrow 0} |\omega ^{\pm }_{j}-\omega _j| = 0. \end{aligned}$$

Moreover, let us denote by $\varvec{X}^\pm _{jh}$ the normalised eigenfunction of (22) associated to $\tau _j^\pm$. If additionally the spectral subspace $\mathcal {E}_{\omega _j}(\mathcal {M})$ lies in the Sobolev space $\mathcal {H}^{r+1}(\Omega )^6$, then there exists a constant $C>0$, independent of *h*, such that

44 $$\begin{aligned} |\omega ^{\pm }_{j}-\omega _j|&\le Ch^{2r},\end{aligned}$$

45 $$\begin{aligned} \inf _{\varvec{X}_j\in \mathcal {E}_{\omega _j}(\mathcal {M})} \Vert \varvec{X}^\pm _{j,h}-\varvec{X}_j\Vert _{{\text {curl}},\Omega }&\le C\, h^r. \end{aligned}$$

Therefore we recover optimal order of convergence under regularity of the eigenfunctions.

This regularity assumption on the corresponding vector spaces can be formulated in different ways in order to suit the chosen algorithm. For the one we have employed here, if we wish to obtain a lower/upper bound for the *j*-eigenvalue to the left/right of a fixed *t* (and consequently obtain approximate eigenvectors) all the vectors of the sum of all eigenspaces up to *j* have to be regular. If by some misfortune an intermediate eigenspace does not fullfill this requirement, then the algorithm will converge slowly. To circumvent this difficulty, the computational procedure can be modified in many ways. For instance, it can be allowed to split iteratively the initial interval, once it is clear that some accuracy can not be achieved after a fixed number of steps. See [3, Procedure 1].

### Order of convergence on a cube

The eigenfunctions of (40) are regular in the interior of a convex domain. In this case, the method of Zimmermann and Mertins for the resonant cavity problem achieves an optimal order of convergence in the context of finite elements.

Let $\Omega =\Omega _{\mathrm {c}}=(0,\pi )^3\subset \mathbb R^3$. The non-zero eigenvalues are

$$\begin{aligned} \omega =\pm \sqrt{l^2+m^2+n^2} \end{aligned}$$

and the corresponding eigenfunctions are

$$\begin{aligned} {\varvec{E}}(x,y,z)= \begin{pmatrix} \alpha _1 \cos (lx) \sin (my) \sin (nz) \\ \alpha _2 \sin (lx) \cos (my) \sin (nz) \\ \alpha _3 \sin (lx) \sin (my) \cos (nz) \end{pmatrix} \quad \forall \underline{\alpha }= \begin{pmatrix} \alpha _1 \\ \alpha _2 \\ \alpha _3 \end{pmatrix} \quad \text{ s.t. } \underline{\alpha }\cdot \begin{pmatrix} l \\ m \\ n \end{pmatrix} =0. \end{aligned}$$

Here $\{l,m,n\}\subset \mathbb {N}\cup \{0\}$ and not two indices are allowed to vanish simultaneously. The vector $\underline{\alpha }$ determines the multiplicity of the eigenvalue for a given triplet (*l*, *m*, *n*). That is, for example, $\omega _1=\sqrt{2}$ (the first positive eigenvalue) has multiplicity 3 corresponding to indices $\{(1,1,0),(0,1,1),(1,0,1)\}$ each one of them contributing to one of the dimensions of the eigenspace. However, $\omega _2=\sqrt{3}$ (the second positive eigenvalue) corresponding to index $\{(1,1,1)\}$ has multiplicity 2 determined by $\underline{\alpha }$ on a plane.

In the present case, we know exactly the number of eigenvalues, counting multiplicity, in a given interval $(t_1,t_2)\subset (0,+\infty )$. Hence using Theorem 16, from $t_1$ we can obtain guaranteed upper bounds for each of the spectral values in this interval and from $t_2$ guaranteed lower bounds. The regularity of the eigenvectors ensures that for a reasonably refined mesh the resulting enclosures do not overlaps (except for multiple eigenvalues).

**Fig. 1:**
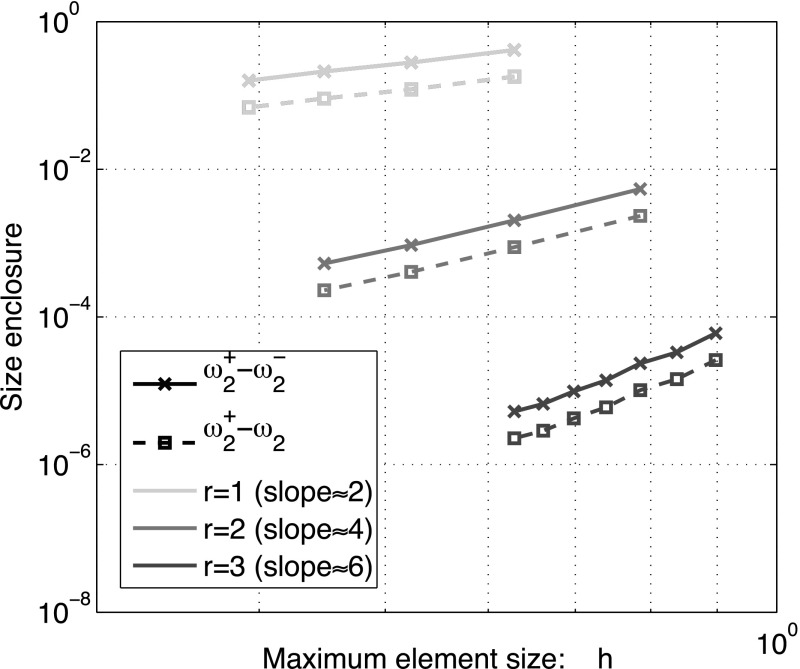
Log–log graph associated to $\Omega _\mathrm {c}$ and $\omega _{2}=\sqrt{3}$. *Vertical axis* upper bound minus lower bound. *Horizontal axis* maximum element size *h*. We have implemented Lagrange elements of order $r=1,2,3$ on a sequence of unstructured meshes. Here we have chosen $t=\frac{\sqrt{2}+\sqrt{3}}{2}$ for the upper bounds and $t=\frac{\sqrt{3}+\sqrt{5}}{2}$ for the lower bounds

In Fig. 1 we have depicted the decrease in enclosure width and absolute error,

$$\begin{aligned} \omega ^+_2-\omega ^-_2 \quad \text {and} \quad \omega ^+_2-\omega _2, \end{aligned}$$

for the computed bounds of the eigenvalue $\omega _2=\sqrt{3}$ by means of Lagrange elements of order $r=1,2,3$. In this experiment we have chosen a sequence of unstructured tetrahedral meshes. The values for the slopes of the straight lines indicate that the enclosures obey the estimate of the form

46 $$\begin{aligned} |\omega ^\pm -\omega | \le c h^{2r}, \end{aligned}$$

which is indeed the optimal convergence rate.

**Fig. 2:**
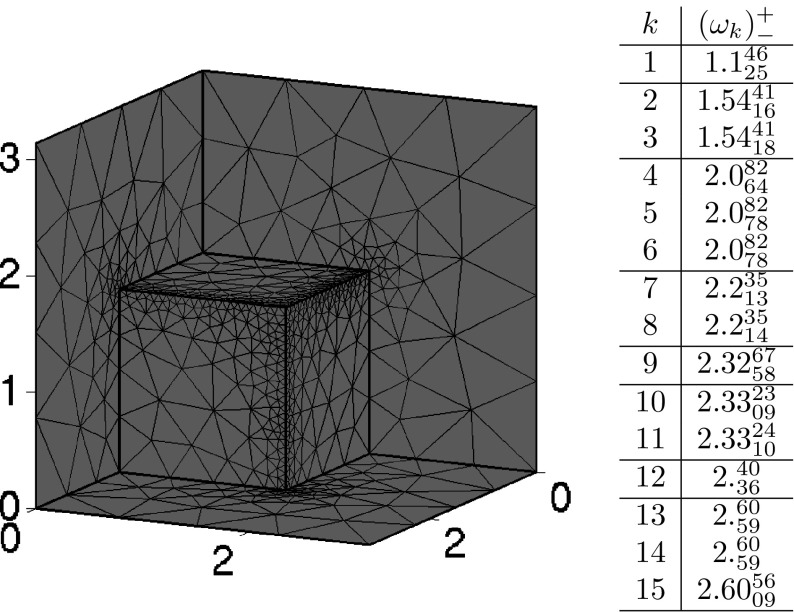
Conjectured enclosures for the spectrum lying on the interval $(0,2\sqrt{2})$ for the Fichera domain $\Omega _{\mathrm {F}}$. Here we have fixed $t=0.2$ to compute the upper bounds and $t=2.8$ to compute the lower bounds. We considered mesh refined at the re-entrant edges as shown on the *left*. The number of $\mathrm{DOF}=208{,}680$

### Benchmark eigenvalue bounds for the Fichera domain

In this next experiment we consider the region $\Omega =\Omega _{\mathrm {F}}=(0,\pi )^3 {\setminus } [0,\pi /2]^3$. Some of the eigenvalues can be obtained by domain decomposition and the corresponding eigenfunctions are regular. For example, eigenfunctions on the cube of side $\pi /2$ can be assembled in a suitable fashion, in order to build eigenfunctions on $\Omega _{\mathrm {F}}$. Therefore the set $\{\pm 2\sqrt{l^2+m^2+n^2}\}$ where not two indices vanish simultaneously certainly lies inside $\sigma (\mathcal M)$. The first eigenvalue in this set is $2\sqrt{2}$.

We conjecture that there are exactly 15 eigenvalues in the interval $(0,2\sqrt{2})$. Furthermore, we conjecture that the multiplicity counting of the spectrum in this interval is

$$\begin{aligned} 1,\,2,\,3,\,2,\,1,\,2,\,1,\,3. \end{aligned}$$

The table on the right of Fig. 2 shows a numerical estimation of these eigenvalues. We have considered a mesh refined along the re-entrant edges as shown on the left side of this figure.

The slight numerical discrepancy shown in the table for the seemingly multiple eigenvalues appears to be a consequence of the fact that the meshes employed are not symmetric with respect to permutation of the spacial coordinates. See [3, Section 6.2] for more details.
